# Predominantly Antibody-Deficient Patients With Non-infectious Complications Have Reduced Naive B, Treg, Th17, and Tfh17 Cells

**DOI:** 10.3389/fimmu.2019.02593

**Published:** 2019-11-15

**Authors:** Emily S. J. Edwards, Julian J. Bosco, Pei M. Aui, Robert G. Stirling, Paul U. Cameron, Josh Chatelier, Fiona Hore-Lacy, Robyn E. O'Hehir, Menno C. van Zelm

**Affiliations:** ^1^Department of Immunology and Pathology, Central Clinical School, Monash University and The Alfred Hospital, Melbourne, VIC, Australia; ^2^The Jeffrey Modell Diagnostic and Research Centre for Primary Immunodeficiencies in Melbourne, Melbourne, VIC, Australia; ^3^Allergy, Asthma and Clinical Immunology Service, Department of Respiratory, Allergy and Clinical Immunology (Research), Central Clinical School, The Alfred Hospital, Melbourne, VIC, Australia

**Keywords:** predominantly antibody deficiency, common variable immunodeficiency, autoimmunity, X-linked agammaglobulinemia, follicular helper T cells, CD21^lo^ B cells, naive T cells, EuroFlow

## Abstract

**Background:** Patients with predominantly antibody deficiency (PAD) suffer from severe and recurrent infections that require lifelong immunoglobulin replacement and prophylactic antibiotic treatment. Disease incidence is estimated to be 1:25,000 worldwide, and up to 68% of patients develop non-infectious complications (NIC) including autoimmunity, which are difficult to treat, causing high morbidity, and early mortality. Currently, the etiology of NIC is unknown, and there are no diagnostic and prognostic markers to identify patients at risk.

**Objectives:** To identify immune cell markers that associate with NIC in PAD patients.

**Methods:** We developed a standardized 11-color flow cytometry panel that was utilized for in-depth analysis of B and T cells in 62 adult PAD patients and 59 age-matched controls.

**Results:** Nine males had mutations in Bruton's tyrosine kinase (BTK) and were defined as having X-linked agammaglobulinemia. The remaining 53 patients were not genetically defined and were clinically diagnosed with agammaglobulinemia (*n* = 1), common variable immunodeficiency (CVID) (*n* = 32), hypogammaglobulinemia (*n* = 13), IgG subclass deficiency (*n* = 1), and specific polysaccharide antibody deficiency (*n* = 6). Of the 53, 30 (57%) had one or more NICs, 24 patients had reduced B-cell numbers, and 17 had reduced T-cell numbers. Both PAD–NIC and PAD+NIC groups had significantly reduced Ig class-switched memory B cells and naive CD4 and CD8 T-cell numbers. Naive and IgM memory B cells, Treg, Th17, and Tfh17 cells were specifically reduced in the PAD+NIC group. CD21^lo^ B cells and Tfh cells were increased in frequencies, but not in absolute numbers in PAD+NIC.

**Conclusion:** The previously reported increased frequencies of CD21^lo^ B cells and Tfh cells are the indirect result of reduced naive B-cell and T-cell numbers. Hence, correct interpretation of immunophenotyping of immunodeficiencies is critically dependent on absolute cell counts. Finally, the defects in naive B- and T-cell numbers suggest a mild combined immunodeficiency in PAD patients with NIC. Together with the reductions in Th17, Treg, and Tfh17 numbers, these key differences could be utilized as biomarkers to support definitive diagnosis and to predict for disease progression.

## Introduction

Predominantly antibody deficiency (PAD) represents the largest group of primary immunodeficiencies (PIDs) and includes up to 70% of all patients (1–5). The hallmark of PAD is a history of severe and recurrent sinopulmonary infections and poor vaccination responses underpinned by impaired B-cell differentiation and antibody production. As such, these patients require lifelong immunoglobulin replacement therapy (IgRT) and prophylactic antibiotics (3, 4).

The archetypical PAD is agammaglobulinemia (6), with patients lacking all serum Ig isotypes as well as circulating B cells. The majority of patients are boys suffering from X-linked agammaglobulinemia (XLA) as a result of mutations in the gene encoding Bruton's tyrosine kinase (*BTK*) (7, 8), an enzyme pivotal in the development of B cells. Autosomal recessive agammaglobulinemia is typically the result of mutations in other components of the pre-B-cell receptor complex or in B-cell transcription factors (9–15).

In a minority of PAD patients with circulating B cells, monogenic defects underpinning clinical phenotype, and pathophysiology have been identified. Most involve B-cell receptor signaling components or molecules required for T: B cell interactions (2, 5, 15–27). With the advancements in genomics, genetic diagnosis is now feasible for 20–30% of patients (28–30). However, for the remainder, diagnosis is typically made by ways of exclusion of clinical and general immunological characteristics (5).

Common variable immunodeficiency (CVID) is the most well-defined PAD (2, 5) and is defined diagnostically by reduced total serum IgG and IgA and/or IgM levels in the presence of impaired vaccination responses and recurrent bacterial infections (31–35). Less well-defined PADs include unclassified hypogammaglobulinemia (HGG), defined by reduced IgG levels but normal IgM and IgA levels; specific polysaccharide antibody deficiency (SpAD), defined by normal serum Ig isotypes but the absence of specific antibody responses to vaccination; and IgG subclass deficiency (IGSCD), defined by normal total IgG levels but reduced levels of one or more IgG subclasses (2, 5, 36).

In addition to recurrent infections and resulting complications, up to 68% of PAD patients suffer from non-infectious complications (NICs), which typically include autoimmunity, autoinflammation, gastrointestinal disease, and lymphoid malignancies (3, 37–43). NICs are most frequent in, but not exclusive to, CVID patients (36, 38, 44). The dominant presentation of NIC can overshadow infectious problems and thereby complicate diagnosis of PAD. Furthermore, NICs are often hard to treat, rendering patients at risk of early morbidity, and high mortality (38, 44, 45).

From the early 2000s, immunophenotyping of the B-cell compartment has been utilized to improve PAD diagnosis and has formed the basis of two classification systems: Freiburg (46) and EUROclass (47). In both strategies, CD19^+^ B cells are delineated using normally low Ig switched memory B-cell (smB) frequencies (CD19^+^CD27^+^IgM^−^IgD^−^) and abnormally high proportions of B cells with reduced CD21 expression (CD21^lo^ B cells) to distinguish subsets. In addition, to these two cell subsets, the EUROclass classification also uses abnormally high frequencies of transitional B cells (CD19^+^CD27^−^CD38^+^) for further subgrouping. Subsequently, more detailed studies have been applied to define immunophenotypes for subgroups in patients with CVID or PAD (36, 48, 49). Despite the identification of clearly distinct phenotypes, these have thus far provided limited prognostic value to predict clinical progression or disease complications. The strongest association found to date is the expansion of CD21^lo^ B cells in patients with splenomegaly (47, 50). In addition, marked reductions in total, Ig switched memory, and marginal zone B cells were found to be associated with splenomegaly (47). Furthermore, the presence of any form of autoimmune disease in CVID patients was associated with expansion of CD21^lo^ B cells (46) and significant reductions in plasmablasts (47) and Ig smBs (37). A proportional expansion of transitional B cells was found to be associated with lymphadenopathy, whereas CVID patients with granulomatous disease presented with significant reductions in Ig class-switched memory and marginal zone B cells (47).

Although PAD is by definition a disease resulting from defective antibody production, multiple studies have demonstrated that disturbances in T and natural killer (NK) cell homeostasis likely contribute to the disease etiology and pathophysiology. Specifically, decreased circulating NK-cell numbers in CVID and XLA patients were found to be associated with severe bacterial infections and granulomas (51, 52). Pronounced CD4 T-cell lymphopenia occurs in some CVID patients (44, 53), and reductions in naive CD4 T cells were associated with splenomegaly (53), autoimmunity, and polyclonal lymphoproliferation. Reductions in naive CD8 T-cell numbers have also been associated with autoimmunity in CVID patients (54). In addition, follicular T helper cells (Tfh), regulatory T cells (Treg), and Th17 cell disturbances have been identified in PAD patients. In particular, XLA patients who lack B-cell follicles have reduced numbers of circulating Tfh cells (54), whereas in CVID patients with autoimmunity and/or splenomegaly, increased proportions of circulating Tfh have been observed (37, 55–57). Furthermore, reduced absolute numbers (54) and frequencies of Treg (58, 59) have been described in CVID patients with autoimmunity and/or splenomegaly, and these reductions are associated with the expansion of CD21^lo^ B cells (59). Th17 cells were also decreased in number and frequency in CVID, paralleled by expansions of CD21^lo^ B cells and activated CD4 T cells with no link to clinical manifestations (60). Finally, interleukin (IL)-2 and interferon gamma (IFN-γ) production by CD4 T cells was higher in patients with hepatomegaly, and chemokine receptor CCR5 known to be expressed on Th1 cells was shown to be higher on CD4 T cells in patients with granulomas (61), implying Th1 bias in these patients.

The many published abnormalities in circulating B and T cells, with several potentially correlating with specific clinical phenotypes, stress the need for high-quality and reproducible immunophenotyping of circulating lymphocytes in PAD patients. The EuroFlow consortium has developed standards for instrument setup and sample preparation (62) and multicolor panels to examine PAD patients (63, 64). We here utilized this expertise to develop a compact 11-color flow cytometry panel that was applied to our cohort of 62 adult PAD patients to identify abnormalities in the B- and T-cell compartments, specifically aiming to discriminate between patients with and without NICs.

## Materials and Methods

### Patients

From November 2015 to June 2019, 62 adult patients with a clinical diagnosis of PAD were enrolled in a low-risk research study to examine their blood leukocyte subsets (projects Alfred Health 109/15 and Monash University CF15/771-2015-0344). All patients consented to the collection of their medical information and a donation of 40 ml of blood. In parallel, 59 adult healthy controls were enrolled in a low-risk reference value study (Monash University project 2016-0289) and consented to collection of basic demographics (age, sex, and history of immunological and hematological diseases) and donation of 40 ml of blood. The study was conducted according to the principles of the Declaration of Helsinki and was approved by local human research ethics committees.

### Assessment of Absolute Numbers of Leukocyte and Lymphocyte Subsets

Absolute numbers of leukocytes were determined using a lyse-no-wash method within 24 h of blood sampling in Vacutainers containing EDTA (BD Biosciences). Fifty microliters of whole blood was added to a TruCount tube (BD Biosciences) together with an antibody cocktail of 20 μl to stain CD3, CD4, CD8, CD16, CD45, and CD56 (Supplementary Figure 1 and Supplementary Tables 1, 2, 5). Following incubation for 15 min at room temperature, 500 μl of 0.155 M NH_4_Cl was added to lyse red blood cells for 15 min. Subsequently, the mixture was stored in the dark at 4°C prior to acquisition on a flow cytometer within 2 h.

### Design of Three Multicolor Tubes for Staining B- and T-Cell Subsets

The design of the tubes was based on the EuroFlow PID antibody panels (64), which had undergone stringent testing and optimization in a multi-laboratory setting. In contrast to the EuroFlow PID initiative, we undertook a research study of adult patients with the intent to examine all cell subsets in all enrolled individuals. Hence, the PID orientation tube (PIDOT) (63, 65) was not included, as the data obtained would be redundant with subsequent lineage-specific analysis. In addition, we had access to 11 fluorescent parameters. This enabled us to merge the EuroFlow pre-GC and post-GC tubes, which share six of the eight markers into one B-cell tube with 12 markers in 10 fluorescent channels (Supplementary Figure 2 and Supplementary Tables 1–3). For eight of the 12 markers, the same reagents were used as in the eight-color EuroFlow panel (64). The same antibody clones were used for CD5, CD21, and CD38, with a different fluorescent label; and for CD19, different clone (SJ25C1) and fluorochrome were used.

Similarly, the EuroFlow SCID/RTE and T-cell tubes were combined into one 11-parameter T-effector tube. Of the 11 markers, seven were identical to those of the EuroFlow protocol (64). For CD3 and HLA-DR, the same clones were used on different fluorochromes, and for CCR7 (CD197), a different clone (G043H7) and another fluorochrome were used. CD45RA was newly inserted in this tube, as this is present in the EuroFlow PIDOT, whereas CD62L was not included owing to redundancy with the CCR7 marker (Supplementary Figure 3 and Supplementary Tables 1–3).

Finally, an 11-color Th-subset tube was designed based on our previous work (66) with the objective to use membrane markers to distinguish Treg (67, 68), helper T-cell subsets (Th) (69, 70), and Tfh (71, 72), and their subsets (73) (Supplementary Figure 4 and Supplementary Tables 1–3). This resulted in a panel of three tubes (B cell, T effector, and T helper) in addition to the TruCount analysis (Supplementary Table 1), and these were run for all patients and controls enrolled in the study.

### Sample Preparation for B-Cell and T-Cell Subset Tubes

To gain detailed insight into the composition of the lymphocyte compartment, including robust identification of small cell populations such as plasma cell subsets, standard operating procedures (SOPs) from the EuroFlow consortium were adopted for acquisition of high cell numbers (1–5 × 10^6^ total nucleated cells) (74–76). The bulk–lysis–stain technique was performed (62, 74). Briefly, samples (up to 2 ml) were diluted in a total volume of 50 ml of an NH_4_Cl hypotonic solution, gently mixed, and incubated for 15 min at room temperature on a roller. Then, nucleated cells were centrifuged and washed twice in phosphate-buffered saline (PBS) containing 0.5% bovine serum albumin (BSA). Subsequently, the surface membrane markers on nucleated cells were stained with the corresponding antibody mixtures.

### Flow Cytometer Setup

All flow cytometry was performed across three instruments in our flow core facility that contained either four lasers (BD LSRII and BD LSRFortessa) or five lasers (BD LSRFortessa X-20) with a nearly identical setup for the shared four lasers (Supplementary Table 4). Instrument setup and calibration were performed using EuroFlow SOPs as previously described in detail (Supplementary Table 5) (62), with in-house optimization for the additional three fluorescent channels (V610, V710, and YG610).

### Data Analysis and Statistics

All data were analyzed with FACS DIVA v8.0.1 (BD Biosciences) and FlowJo v10 software packages (FlowJo, LLC). Reference ranges were defined as being within the 5th and 95th percentiles of absolute cell numbers from our 59 adult controls. Statistical analysis for multiple-group comparison was performed with the non-parametric Kruskal-Wallis test. If significant, pairwise comparisons were made with the non-parametric Mann-Whitney *U* test. Statistical analysis of sampling distributions was assessed with the chi-square test. For all tests, *p* < 0.05 was considered significant.

## Results

### Clinical and Immunological Features of Predominantly Antibody Deficiency Patients

Sixty-two PAD patients were recruited in a prospective research study from a teaching hospital in Melbourne, Australia. Median age of the patients was 43 years (range, 18–82 years), and 34 were female (Table 1). CVID was the most common clinical diagnosis in 52% of all patients, followed by 21% with HGG, 16% with agammaglobulinemia, 9% with SpAD, and 2% with IGSCD. Of the 10 patients diagnosed with agammaglobulinemia, nine were male and genetically confirmed to have XLA (Table 1 and Supplementary Tables 6, 7). The other 53 patients did not undergo any genetic testing.

**Table 1:** Demographics, clinical details, and diagnostic results of the patients in this study.

	**Healthy controls** **(*n* = 59)**	**All patients** **(*n* = 62)**	**PAD–NIC** **(*n* = 23)**	**PAD+NIC** **(*n* = 30)**	**XLA** **(*n* = 9)**
**DEMOGRAPHICS**					
Median age (years; range)					
At inclusion	30 (20–71)	43 (18–82)	46 (19–73)	44 (23–82)	24 (18–59)
At diagnosis	N/A	35 (2 months to 74 years)	45 (18–73)	36 (12–74)	9 (2 months to 13 years)
Female sex (%)	33 (58%)	34 (55%)	14 (61%)	20 (67%)	0
Clinical diagnosis					
Agammaglobulinemia (%)	0	10 (16%)	1 (4%)	0	9 (100%)
CVID (%)	0	32 (52%)	9 (39%)	23 (77%)	0
HGG (%)	0	13 (21%)	7 (30%)	6 (20%)	0
IGSCD (%)	0	1 (2%)	1 (4%)	0	0
SpAD (%)	0	6 (9%)	5 (22%)	1 (3%)	0
**IMMUNOLOGICAL PRESENTATION**					
Decreased serum immunoglobulin levels					
IgG (%)	N/A	40/54 (74%)	14/23 (61%)	26/30 (87%)	0/1^#^
IgA (%)	N/A	46/61 (75%)	15/23 (65%)	24/30 (80%)	8/8 (100%)
IgM (%)	N/A	34/61 (56%)	11/23 (48%)	15/30 (50%)	8/8 (100%)
Impaired vaccination responses (%)	N/A	25/30 (83%)	12/16 (75%)	12/14 (86%)	N/A^#^
Reduced cell numbers					
B cells (%)	N/A	24 (39%)	3 (13%)	12 (40%)	9 (100%)
T cells (%)	N/A	17 (27%)	5 (22%)	11 (37%)	2 (22%)
**TREATMENT**					
IgRT at sampling (%)	N/A	46 (74%)	11 (48%)	25 (83%)	9 (100%)
IgRT started after inclusion (%)	N/A	12 (19%)	7 (30%)	5 (17%)	N/A
Immunomodulators^*^ (%)	N/A	8 (13%)	3 (13%)	4 (13%)	1 (11%)

*Reference ranges: IgG, 6.1–16.2 g/L; IgA, 0.85–4.99 g/L; IgM, 0.35–2.42 g/L; B cells, 97–614 cells/μl; T cells, 830–2,430 cells/μl*.

*(B- and T-cell reference ranges were derived from the 5th and 95th percentiles of our healthy controls)*.

# *Serum IgG levels and vaccination responses not assessed owing to historic nature of disease*.

* *On immunomodulators within 6 months prior to blood sampling*.

*PAD, predominantly antibody deficiency; NIC, non-infectious complications; XLA, X-linked agammaglobulinemia; CVID, common variable immunodeficiency; HGG, hypogammaglobulinemia; IGSCD, IgG subclass deficiency; SpAD, specific polysaccharide antibody deficiency; IgRT, immunoglobulin replacement therapy; IGSCD, IgG subclass deficiency. A subset of patients has been reported in a recent publication (77)*.

All patients presented with infectious manifestations (Table 2 and Supplementary Table 6), and these were generally confined to the respiratory tract: sinusitis (77%), pneumonia (61%), and otitis (32%) with frequent complications of bronchiectasis (27%). Infections of the gastrointestinal tract and other sites, such as the prostate and bone, were less frequently involved (19%). Of all 62 patients, 30 (48%) presented with at least one NIC, with autoimmunity being the most frequent (29%), followed by gastrointestinal (18%), and granulomatous diseases (5%; Table 2). At the time of inclusion in the study, 46 (74%) patients were treated with IgRT. A further 12 (19%) newly diagnosed patients commenced IgRT directly after inclusion. Eight patients (13%) had been prescribed immunomodulators within 6 months prior to inclusion (Table 1). Three patients were treated with immunomodulators for asthma or for IgRT tolerability, which were deemed unrelated to their PAD, and thus, these patients were defined as PAD–NIC.

**Table 2:** Complications in patients with predominantly antibody deficiency.

	**All patients** **(*n* = 62)**	**PAD–NIC** **(*n* = 23)**	**PAD+NIC** **(*n* = 30)**	**XLA** **(*n* = 9)**
**INFECTIOUS COMPLICATIONS**				
URTI (%)	49 (79%)	17 (74%)	24 (80%)	8 (89%)
Sinusitis (%)	48 (77%)	16 (70%)	24 (80%)	8 (89%)
Otitis (%)	20 (32%)	7 (30%)	7 (23%)	6 (67%)
LRTI (%)	49 (79%)	17 (74%)	25 (83%)	8 (89%)
Bronchitis (%)	8 (13%)	4 (17%)	4 (13%)	0
Pneumonia (%)	38 (61%)	12 (52%)	20 (67%)	7 (78%)
Bronchiectasis (%)	17 (27%)	4 (17%)	8 (27%)	5 (56%)
Gastrointestinal (%)	4 (6%)	0	4 (13%)	0
Giardia (%)	4 (6%)	0	4 (13%)	0
Other sites^*^	8 (13%)	1 (4%)	6 (20%)	1 (11%)
**NON-INFECTIOUS COMPLICATIONS**				
GLILD (%)	1 (2%)	0	1 (3%)	0
Autoimmunity (total) (%)	18 (29%)	0	18 (60%)	0
Musculoskeletal (%)	5 (8%)	0	5 (17%)	0
Cytopenia (%)	6 (10%)	0	6 (20%)	0
Endocrine (%)	3 (5%)	0	3 (10%)	0
Splenomegaly (%)	4 (6%)	0	4 (13%)	0
Lymphadenopathy (%)	1 (2%)	0	1 (3%)	0
Gastrointestinal disease total) (%)	11 (18%)	0	11 (37%)	0
Enteropathy (%)	10 (16%)	0	10 (33%)	0
Colitis (%)	2 (3%)	0	2 (7%)	0
Granulomatous disease (%)	3 (5%)	0	3 (10%)	0
Malignancy (%)	2 (3%)	0	2 (7%)	0
Solid Organ (%)	2 (3%)	0	2 (7%)	0
Hematological (%)	0	0	0	0^#^

*PAD, predominantly antibody deficiency; PAD–NIC, PAD without non-infectious complications; PAD+NIC, PAD with non-infectious complications; XLA, X-linked agammaglobulinemia; GLILD, granulomatous-lymphocytic interstitial lung disease*.

* *Other includes osteomyelitis, pertussis, prostatitis, and systemic viral infection*.

# *One XLA patient developed an acute precursor-B-cell leukemia 6 months after inclusion in this study (78)*.

Serum IgG levels prior to commencement of IgRT were obtained from medical records of 47 patients (Table 1), and in 40/47 (85%), these were below the normal range. Serum IgA and IgM concentrations were available for 61 patients, and of these, 46 (75%) had reduced IgA and 34 (56%) had reduced IgM. The results of vaccination responses in most cases to polysaccharide pneumococcal vaccine were documented for 30 patients and impaired in 25 (83%). B- and T-cell numbers were below the normal range (<5th percentile of our healthy control cohort) for 39 and 27% of all patients, respectively. A total of 11% of patients had a reduction of both B- and T-cell numbers (Table 1). B-cell numbers were below the normal range for all nine XLA patients, with two patients (22%) also having reduced T-cell numbers.

For further immunological analysis, the PAD patient cohort was divided into three groups: 9 patients with genetically diagnosed XLA, 23 PAD patients without NICs (PAD–NIC), and 30 with NICs (PAD+NIC). The nine XLA patients presented with infectious complications only, and these included respiratory infections (89%). Five XLA patients (56%) had evidence of bronchiectasis; this incidence was 2-fold higher than in other PAD groups (17% in PAD–NIC; 27% in PAD+NIC). Of the 32 CVID patients, 23 (72%) presented with one or more NICs (Table 2), whereas only a minority of patients diagnosed with HGG or SpAD presented with NIC. By definition, PAD+NIC patients displayed a more severe and complex clinical phenotype (Table 2) and were diagnosed at a younger age than were PAD–NIC patients (median age at diagnosis: 3 vs. 45 years).

All classifications of patients were undertaken alongside those of 59 healthy controls (median age 30 years and 55% females). The healthy controls and the non-XLA PAD patients were classified according to the Freiburg and EUROclass definitions (47, 79) on the basis of their B-cell phenotypes (Table 3). According to the Freiburg classification, 95% of controls had normal frequencies of smBs and no increases in CD21^lo^ B cells (group II), with the remaining 5% having low smB (Ib). The majority of all PAD patients (29; 55%) had reduced smB frequencies (Ia/Ib; *p* < 0.0001 vs. controls). Seven PAD patients had increased frequencies of CD21^lo^ B cells (Ia), and the majority of these patients (*n* = 5) were in the PAD+NIC group. According to the EUROclass scheme, all controls had normal smB and CD21^lo^ B-cell frequencies (Table 3). Of all PAD patients, 12 (22%) had reduced smB frequencies and 13 (25%) had increased CD21^lo^ B-cell frequencies. Slightly more PAD+NIC patients had reduced smB and increased CD21^lo^ B cells than had PAD–NIC, but these differences were not significant (CD21^lo^ expansion, *p* = 0.06).

**Table 3:** Classification of PAD patients according to the Freiburg and EUROclass definitions.

	**Healthy controls** **(*n* = 59)**	**All PAD^a^** **(*n* = 53)**	**PAD–NIC** **(*n* = 23)**	**PAD+NIC** **(*n* = 30)**
**FREIBURG**				
Ia (smB−21lo)	0	7 (13%)	2 (9%)	5 (16%)
Ib (smB−21norm)	3 (5%)	22 (42%)	8 (36%)	14 (47%)
II (smB+21lo or norm)	56 (95%)	24 (45%)	13 (55%)	11 (37%)
**EUROCLASS**				
B–	0	3 (6%)	2 (9%)	1 (3%)
B+smB−21normTrnorm	0	5 (9%)	3 (13%)	2 (7%)
B+smB−21normTrhi	0	1 (2%)	0	1 (3%)
B+smB−21loTrnorm	0	5 (9%)	0	5 (17%)
B+smB−21loTrhi	0	1 (2%)	0	1 (3%)
B+smB+21norm	59 (100%)	31 (58%)	15 (65%)	16 (53%)
B+smB+21lo	0	7 (14%)	3 (13%)	4 (14%)

*PAD, predominantly antibody deficiency; NIC, non-infectious complications; smBs, switched memory B cells, 21, CD21; Tr, transitional B cells*.

*Freiburg classification undertaken on patients with ≥1% B cells in lymphocyte gate; definitions (79): smB–, <0.4% CD28^+^IgD^−^ B cells within lymphocytes; 21lo, ≥20% B cells are CD21^lo^*.

*EUROclass definitions (47): B–, <1% of lymphocytes; smB–, <2% of B cells; 21^lo^, ≥10% B cells are CD21^lo^; Trhi, >9% of B cells are transitional (CD38^hi^CD27^−^)*.

a *Excluding patients with a clinical diagnosis of X-linked agammaglobulinemia (XLA), as these were all B cell negative*.

Overall, our patient cohort is diverse in clinical and immunological presentations, in line with previously reported cohorts of adult PAD (38, 44). Importantly, PAD–NIC and PAD+NIC groups are seemingly different in their immunological profiles. With almost equally large PAD–NIC and PAD+NIC groups and a substantial group of genetically diagnosed XLA patients, this cohort is well-suited to examine immunological differences that associate with the presence of NICs.

### Reduced Numbers of Lymphocytes in Predominantly Antibody Deficiency Patients

As a large fraction of the non-XLA PAD patients had B- and/or T-cell numbers below the normal range of controls (Table 1), we first examined the numbers of leukocyte and lymphocyte subsets in our patient groups (Supplementary Figures 1, 5 and Supplementary Table 3). Circulating numbers of granulocytes and monocytes in both PAD–NIC and PAD+NIC were similar to those of controls, whereas these were significantly increased in XLA patients (Supplementary Figures 5A,B). In contrast, total lymphocytes, and NK cells were significantly reduced in both PAD groups as compared with controls, whereas these were normal in the XLA group (Supplementary Figures 5C,D).

### Reduced Total, Naive, and IgM Memory B Cells in Predominantly Antibody Deficiency Patients With Non-infectious Complications

**Figure 1:**
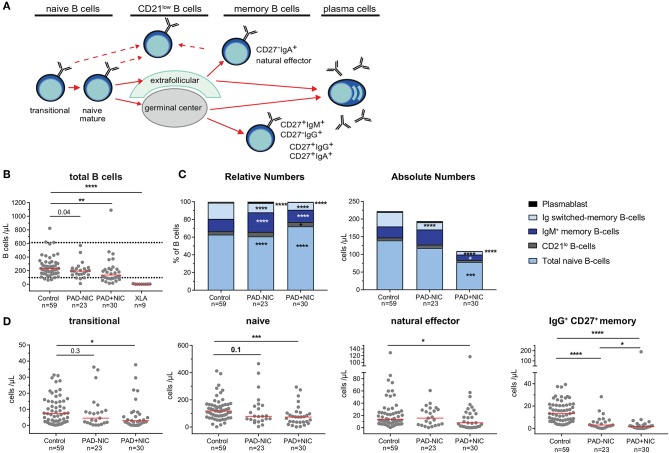
B-cell abnormalities in PAD patients. **(A)** Schematic overview of naive and memory B-cell subsets. **(B)** Absolute numbers of total B cells. Horizontal dotted lines represent the 5th and 95th percentiles of the healthy control group and were used as reference ranges. **(C)** Relative numbers and absolute numbers of B-cell subsets. **(D)** Absolute numbers of transitional, naive, natural effector, and IgG^+^ CD27^+^ memory B cells. For gating strategy and population definitions, see Supplementary Figure 2 and Supplementary Table 3. PAD, predominantly antibody deficiency; NIC, noninfectious complications. Statistics were performed with the Kruskal–Wallis test, followed by Mann–Whitney tests for pairwise comparisons. *p < 0.5, **p < 0.01, ***p < 0.001, and ****p < 0.0001.

Further detailed analysis of the B-cell compartment was restricted to the 59 controls and the 53 non-XLA PAD patients, as B cells were completely absent in the XLA patients (Figure 1A). Numbers of circulating B cells were significantly reduced in the PAD+NIC, but not in the PAD–NIC group (Figure 1B). Within the peripheral B-cell compartment, frequencies of IgM memory, Ig switched memory, and plasmablasts were significantly lower in both PAD–NIC and PAD+NIC compared with controls (Figure 1C). Frequencies of naive B cells were increased in both patient groups. However, when expressed as absolute numbers, these were actually normal in PAD–NIC and significantly reduced in PAD+NIC (Figures 1C,D and Supplementary Figure 6A). IgG and IgA smBs remained reduced in terms of absolute numbers in both PAD groups. In contrast, absolute numbers of IgM memory B cells were normal in PAD–NIC whereas reduced in PAD+NIC (Figures 1C,D and Supplementary Figures 6A,B). Finally, CD21^lo^ B-cell frequencies were increased in the PAD+NIC group, but the absolute numbers of circulating CD21^lo^ B cells were normal (Figure 1C).

Taken together, both PAD–NIC and PAD+NIC have severely reduced numbers of Ig smBs. In addition, as a group, PAD+NIC patients have reduced numbers of circulating total, naive, and IgM memory B cells.

### Reduced Naive CD4 and CD8 T Cells in Predominantly Antibody Deficiency Patients

**Figure 2:**
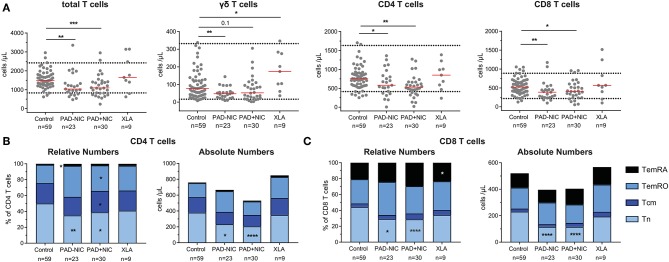
Reduced naive T-cell numbers in PAD patients. **(A)** Absolute numbers of total T cells, γδ T cells, CD4 T cells, and CD8 T cells. Horizontal dotted lines represent the 5th and 95th percentiles as calculated in healthy controls. Relative and absolute numbers of **(B)** CD4 T-cell subsets and **(C)** CD8 T-cell subsets. Naive (Tn; CCR7^**+**^CD45RO^−^), central memory (Tcm; CCR7^**+**^CD45RO^**+**^), effector memory (TemRO; CCR7^−^CD45RO^−^), and effector memory CD45RA revertant (TemRA; CCR7^−^CD45RO^−^). For gating strategy and population definitions, see Supplementary Figure 3 and Supplementary Table 3. PAD, predominantly antibody deficiency; NIC, non-infectious complications. Statistics were performed with the Kruskal–Wallis test, followed by Mann–Whitney tests for pairwise comparisons. *p < 0.5, **p < 0.01, ***p < 0.001, and ****p < 0.0001.

In addition to the lymphocyte, NK-cell, and B-cell abnormalities, the PAD–NIC and PAD+NIC groups had significantly lower numbers of circulating T cells than had controls. These concerned all three major lineages: TCRγδ T cells in PAD–NIC and both CD4 and CD8 T cells in both PAD groups. In contrast, only two XLA patients had reduced total T-cell numbers (Figure 2A).

**Figure 3:**
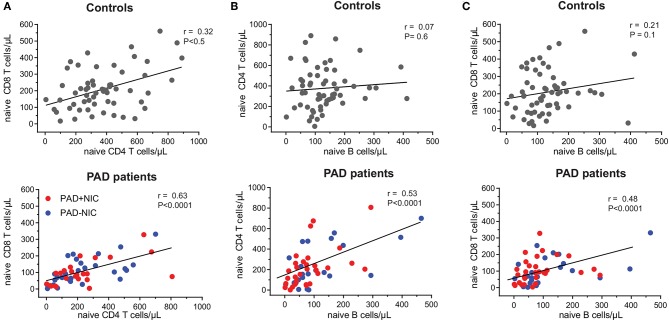
Correlation between naive B- and T-cell numbers in healthy controls and PAD patients. Correlation between absolute numbers of **(A)** naive CD4 and naive CD8 T cells, **(B)** naive B and naive CD4 T cells, and **(C)** naive B and naive CD8 T cells were assessed in healthy controls and PAD patients. Trend lines depict linear correlations for controls (top row; *n* = 59) and total group of non-XLA PAD patients (bottom row = 53). PAD, predominantly antibody deficiency; NIC, non-infectious complications; XLA, X-linked agammaglobulinemia. Statistics were performed using Spearman's rank correlation.

To examine the nature of the T-cell abnormalities, we delineated the CD4 and CD8 lineages into CCR7^+^CD45RO^−^ naive (Tn), CCR7^+^CD45RO^+^ central memory (Tcm), CCR7^−^CD45RO^+^ effector memory (TemRO), and CCR7^−^CD45RO^−^ effector memory (TemRA) T cells (Supplementary Figure 3 and Supplementary Table 3). Frequencies and absolute numbers of naive CD4 and CD8 T cells were reduced in both PAD–NIC and PAD+NIC, but not in XLA patients (Figures 2B,C). To examine the association between naive CD4 and naive CD8 T-cell counts, we performed correlation analysis of these in both the healthy control and the patient cohorts. This revealed a reasonable positive correlation for controls (*r* = 0.32; *p* < 0.05) and strong correlation for total group of 53 non-XLA PAD patients (*r* = 0.63; *p* < 0.0001; Figure 3A). As PAD+NIC patients also had reduced naive B cells, we examined their association with naive CD4 and CD8 T cells. No correlations were found in the control group, but naive B-cell numbers were positively correlated with naive CD4 T cells (*r* = 0.53; *p* < 0.0001) and naive CD8 cells (*r* = 0.48; *p* < 0.0001; Figures 3B,C).

PAD+NIC had increased frequencies of CD4 Tcm and TemRO, but as a result of the reduced total CD4 T cells, these were normal in terms of numbers (Figure 2B). Early-, intermediate-, and late-differentiated TemRO and TemRA cells were distinguished by the progressive loss of CD27 and/or CD28 (80) (Supplementary Figure 3). Within the CD4 TemRO compartment, numbers of early-differentiated cells were increased in PAD–NIC and XLA whereas reduced in PAD+NIC. Intermediate-TemRO cell numbers were reduced in PAD–NIC, and late-TemRO numbers were increased in XLA (Supplementary Figure 7A). Intermediate-TemRA numbers were reduced in PAD+NIC, but proportions were unchanged (Supplementary Figure 7B). CD8 TemRO numbers were not different between patients and controls (Supplementary Figure 7C), whereas CD8 TemRA early and intermediate cell subsets were significantly reduced in all three patient groups (Supplementary Figure 7D).

Overall, both PAD–NIC and PAD+NIC patients exhibit a marked reduction in circulating T cells, mainly as a result of reductions in the naive CD4 and CD8 T-cell subsets.

### Altered Treg and Th Cell Composition in Predominantly Antibody Deficiency Patients With Non-infectious Complications

**Figure 4:**
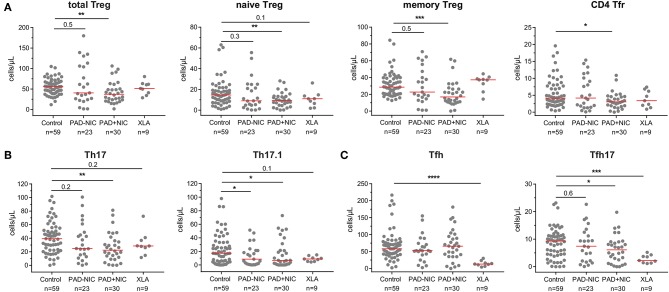
Reduced Treg and helper T-cell numbers in PAD patients with non-infectious complications. **(A)** Total Treg, naive Treg, memory Treg, and CD4 Tfr cells. **(B)** Th17 and Th17.1 cells. **(C)** Total Tfh and Tfh17 cells. All numbers represent cells per microliter of blood. For gating strategy and population definitions, see Supplementary Figure 4 and Supplementary Table 3. PAD, predominantly antibody deficiency; NIC, non-infectious complications. Statistics were performed with the Kruskal–Wallis test, followed by Mann–Whitney tests for pairwise comparisons. *p < 0.5, **p < 0.01, ***p < 0.001, and ****p < 0.0001.

To analyze whether Th numbers were altered in PAD patients with and without NIC, we delineated Treg, Th, Tfh, and their subsets (Supplementary Table 3 and Supplementary Figure 4). Total Treg numbers and their respective naive and memory subsets were significantly lower in PAD+NIC than in controls, whereas these were not changed in PAD–NIC and XLA patients (Figure 4A). The subset of follicular regulatory T(fr) cells was also specifically decreased in PAD+NIC patients (Figure 4A). Within the Th cells, four subsets were defined (i.e., Th1, Th2, Th17, and Th17.1), which are each associated with responses to distinct types of pathogens. No alterations were observed for Th1 (bacterial and viral pathogens) or Th2 cells (extracellular pathogens; data not shown). In contrast, Th17-cell numbers (bacterial and fungal pathogens) were specifically reduced only in PAD+NIC as compared with controls (*p* = 0.005). Finally, numbers of Th17.1 cells (IL-17 and IFN-γ double producers) were reduced in both PAD–NIC and PAD+NIC, but not in XLA patients (Figure 4B).

Finally, we enumerated Tfh numbers, as these are critical for providing help to B cells in germinal center responses. Absolute numbers of Tfh cells were not different between controls, PAD–NIC, and PAD+NIC, whereas these were significantly reduced in XLA patients (*p* ≤ 0.0001). Within the total Tfh population, a similar distinction of four subsets was made as for the Th subsets: Tfh1, Tfh2, Tfh17.1, and Tfh17 (73). Of these, only Tfh17 was significantly reduced in the PAD+NIC and XLA groups as compared with controls (Figure 4C).

Taken together, the CD4 T-cell compartment was most severely affected in PAD+NIC patients with significantly reduced Treg, Th17, Th17.1, and Tfh17 numbers.

### Misinterpretation of Relative Numbers in the Context of Reduced Absolute Cell Numbers

**Figure 5:**
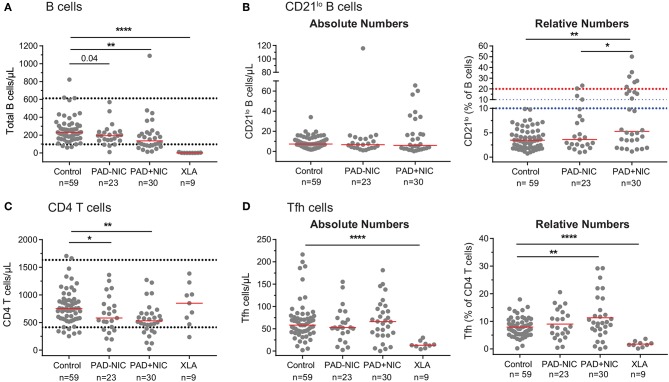
Pitfalls in interpretation of relative expansions of cell subsets in the context of reduced absolute total cell numbers. **(A)** Absolute numbers of total B cells. Horizontal dotted lines represent the 5th and 95th percentiles from the healthy controls. **(B)** Absolute numbers and relative frequencies of CD21^lo^ B cells. Horizontal dotted lines denote the upper thresholds for CD21^lo^ expansions in the Freiburg (red) and EUROclass (blue) classification schemes. **(C)** Absolute numbers of total CD4 T cells. **(D)** Absolute numbers and relative frequencies of Tfh cells. For gating strategies, see Supplementary Figure 2 (CD21^lo^ B cells) and Supplementary Figure 4 (Tfh cells). PAD, predominantly antibody deficiency; NIC, non-infectious complications. Statistics were performed with the Kruskal–Wallis test, followed by Mann–Whitney tests for pairwise comparisons. *p < 0.5, **p < 0.01, and ****p < 0.0001.

Similar to previous reports, we observed in our cohort that PAD+NIC patients had a significantly increased proportion of CD21^lo^ B cells (Figures 1B, 5). However, when expressed as absolute numbers of cells per microliter blood, CD21^lo^ B cells were normal in our cohort (Figure 5B). In fact, the increased proportion of CD21^lo^ B cells was the result of a reduction in total B-cell numbers in the PAD+NIC group. Thus, the increased proportion of CD21^lo^ B cells did not reflect an increase in this subset but rather a decrease of other, mainly naive B cells. In parallel, we confirmed previous reports (37, 55, 56, 81) of increased proportions of Tfh in the PAD+NIC group (Figure 5). However, absolute numbers of Tfh were normal in the context of reduced total CD4 T cells (Figures 5C,D). Thus, the relative expansion does not reflect an abnormality in Tfh but rather is the result of abnormally low total T cells, mainly due to the reduction in the naive subset.

## Discussion

Here we present the results of a standardized approach for extensive immunophenotyping of the B- and T-cell compartments in patients with PAD. Irrespective of the presence of NIC, PAD patients show reduced Ig smBs, Th17.1 cells, and naive CD4 and CD8 T cells. In addition, the group of patients with NICs has severe reductions in total B-cell, Th17, Treg, and Tfh17 numbers. Finally, expansions of CD21^lo^ B-cell and Tfh-cell frequencies are the product of reduced total B-cell and T-cell numbers, and not of absolute increases. Thus, our results demonstrate the importance of structured immunophenotypic analysis with the inclusion of absolute cell numbers to delineate affected cell types in PAD patients with NICs.

We here examined a cohort of 62 PAD patients. These included nine genetically diagnosed XLA patients, who were all diagnosed in early childhood and, prior to inclusion into our study, suffered from infectious complications only. Although the XLA group was by far the smallest, it does represent a homogenous group of patients with a B-cell intrinsic defect to be used as patient controls for our PAD–NIC and PAD+NIC immunophenotying. The vast majority of the other PAD patients were diagnosed with CVID (32; 52%) or HGG (13; 21%). This is in line with previous reports from teaching hospitals and is most likely reflective of the skewed population seen in tertiary care (36, 38, 44, 49). In our cohort, the most frequent infections were of the respiratory tract (13%, upper respiratory tract infection [URTI] only; 21% lower respiratory tract infection [LRTI] only; 66% URTI plus LRTI), with the most prevalent NIC being autoimmunity (60%) (38, 44, 82–85), which is reflective of other clinical studies. Furthermore, the significantly higher incidence of NIC in patients with a CVID diagnosis (72%) vs. all other non-XLA PAD (35%; *p* = 0.01) is similar to that of previous reports (36, 38, 44, 83). In terms of patient numbers, it falls short of multi-institute cohort analyses of clinical and basic immunological features (38, 44, 83, 86). However, our cohort of 62 patients relates well to other immunophenotyping studies (37, 46, 48, 51–53, 79, 87) and is larger than most studies (37, 46, 55, 58–61, 79). Moreover, the division into almost equally sized groups of PAD–NIC (*n* = 23) and PAD+NIC (*n* = 30) and a genetically defined XLA patient control group (*n* = 9) is well-suited for examination of common and NIC-associated abnormalities. The high prevalence of NICs in adult PAD patients in our study likely associates with later onset of symptoms (86) and delayed diagnosis in these patients (38, 44). The clinical and immunological presentations of our patient cohort are diverse, in line with previously published studies. Thus, this cohort is highly representative for the in-depth analysis of immunological differences that segregate with the presence of NICs.

In our study, reduced B-cell numbers segregated with the presence of NICs. Further in-depth examination of the B-cell compartment revealed significant reductions in Ig smBs and plasmablasts in both PAD groups, which was in line with a recent PAD study (48). However, unlike previous reports (37, 47), in our cohort, these abnormalities did not segregate with NICs such as splenomegaly, granulomatous disease, and autoimmunity. In contrast, we observed specific reductions in serum IgM levels and IgM memory B-cell numbers in PAD+NIC patients. As IgM has a role in preventing autoimmunity by promoting phagocytic clearance of cell debris including autoantigen (88, 89), it is possible that this defect contributes to the autoimmune pathophysiology in patients with PAD+NIC.

In addition to the reduced memory B cells, naive B cells were significantly reduced in the PAD+NIC group. As naive B cells form the largest proportion of total B cells, this reduction is mostly responsible for the reduced total B-cell numbers. The cause of reduced B-cell numbers is unclear and could be related to either reduced production from bone marrow precursors or reduced survival in the periphery. Gene defects underlying either of these processes have been reported in patients with an antibody deficiency syndrome (2). Importantly, the reductions in naive B cells correlated with reduced naive CD4 and CD8 T cells (see *Discussion* below) and could be part of a more general lymphocyte production and/or survival defect that contributes to disease pathology. Finally, the reduction in naive B-cell numbers highlights the importance of measuring absolute cell numbers, as this will affect proportions of other cell subsets (e.g., CD21^lo^ B cells; see below) and lead to misinterpretation of indirect findings.

We here show that the expansion of CD21^lo^ B cells, which is reportedly associated with autoimmunity and splenomegaly in CVID patients (46, 47), was only observed in PAD+NIC patients when presented as frequency of total B cells, but not in terms of absolute cell numbers. Expansion of this B-cell subset has also been identified in numerous chronic infections and autoimmune and granulomatous diseases. Specifically, increased frequencies and numbers of CD21^lo^ B cells have been observed in Crohn's disease (90), Sjögren's syndrome (91), and poor HIV controllers (92). In addition, increased CD21^lo^ B-cell frequencies have also been identified in rheumatoid arthritis (RA) (93, 94), scleroderma (94), systemic lupus erythematosus (SLE) (95), and multiple sclerosis (MS) (96). Similar to PAD+NIC, absolute numbers of CD21^lo^ B cells are normal in RA (93), and the increased frequencies of CD21^lo^ B cells were the result of reduced naive and memory B-cell numbers (93). This segregates PAD+NIC and RA from Crohn's disease, Sjögren's syndrome, and HIV in which the absolute numbers of CD21^lo^ B cell are increased and might be indicative of distinct pathophysiologies.

Total CD4 and CD8 T-cell numbers were significantly lower in both PAD–NIC and PAD+NIC groups as a result of a reduction in the naive subsets. In contrast to other studies where naive T-cell deficiencies associated with NICs such as splenomegaly (53, 54), this link was not observed in our study. In addition, as memory T-cell numbers were unaffected, we postulate that decreased total CD4 and CD8 T-cell numbers are as a result of smaller numbers of naive T cells. Although PAD has long been considered to result from disturbances in B-cell homeostasis, it is becoming more evident that defective T-cell responses also play a role in disease pathogenesis. In particular, reductions in naive CD4 and CD8 T cells will likely impair immunity in primary infection and vaccination, thus rendering patients more prone to severe and often lethal infections, as shown in studies of aging and HIV infection (97–100).

Further dissection of the CD4 T-cell compartment in our cohort revealed abnormalities in Treg, Th, and Tfh cells. Treg-cell numbers were specifically low in PAD+NIC patients, in line with previous reports (54, 58, 59). This concerned both thymus-derived, naive Treg, and peripherally induced memory Treg. It is very tempting to speculate that the Treg deficiency contributes to the autoimmune pathology, similar to what has been proposed for patients with type 1 diabetes, MS, and SLE (101–106). Still, it remains unclear what causes the reduction and how this contributes to NIC. Potentially, the reduction is related to the reduced total naive T cells and might be reflective of either impaired production from the thymus, or increased maturation into effector memory cells as a result of the high infectious pressure in PAD patients.

Although the precise role of Th17.1 cells in immunity has not yet been fully elucidated, decreased Th17.1 counts have been linked to increased susceptibility to bacterial infection in other immunocompromised patients, including those with hyper-IgE syndrome and HIV/AIDS (107–112). In addition to the function of Th17 cells in controlling infections, these cells have been shown to promote antibody production by B cells (60). Thus, reductions in these cell numbers might contribute to the impaired antibody responses in PAD patients.

In line with previous reports, we observed increased frequencies of Tfh cells in patients with NIC (37, 55, 57). However, we also determined the absolute Tfh numbers and found that these were similar to those of controls. Thus, rather than a role of increased Tfh cells in CVID and autoimmunity (37, 55, 57, 113–115), it might be an altered distribution of Tfh subsets that contributes to disease pathology. Importantly, in PAD+NIC patients, the most potent B-cell helpers, Tfh17 cells, are significantly reduced. This could potentially link the inefficient antibody responses to pathological B cell responses, driven by, for example, the other Tfh cell subsets.

In our study, we utilized membrane markers only for the delineation of Th, Tfh, and Treg cells. In literature, multiple phenotypic definitions have been used to assess Th cells (cytoplasmic IFN-γ, IL-4, IL-10, and IL-17), Tfh cells (CD4^+^CXCR5^+^, CD4^+^CXCR5^+^PD-1^+^, or CD4^+^CXCR5^+^ICOS^+^), and Treg cells (CD4^+^CD25^+^CD127^lo/−^ or CD4^+^CD25^+^FoxP3^+^) (71, 72, 116, 117). Although intracellular FoxP3 and cytokine expression represents the gold standard protocols for the delineation of Treg and Th subsets, respectively, these protocols are laborious and not easily amenable to high-throughput analyses. The surface markers utilized to define Treg and Th subsets in our panel have previously been demonstrated to correlate well with intracellular FoxP3 (116) and cytokine expression (117, 118). Therefore, we are convinced that the gating strategies we applied were highly specific. Without the need for *in vitro* activation and/or cytoplasmic staining, our protocol is more straightforward, making it quicker and more easily scalable for adoption in diagnostic laboratories.

Here, we have identified B- and T-cell biomarkers associated with NICs in patients with PAD. Humoral and cellular deficiencies have previously been associated with aging contributing to susceptibility to infection and NIC development including autoimmunity and cancer. This is particularly the case for declining numbers of naive B and T cells (74, 119–121). Thus, it could be suggested that PAD–NIC represent a precursor group whom with aging will develop NICs. However, the lower median age at diagnosis of the PAD+NIC group (25 vs. 45 years in PAD–NIC) suggests that it is more likely that the PAD–NIC and PAD+NIC patients suffer from distinct pathophysiologies.

CVID demonstrates intrinsic clinical, immunological, and genomic heterogeneity complicating diagnosis resulting in decreased overall survival rates (82, 122, 123). Over the past 5 years, criteria utilized to define CVID have been expanded to include total and naive CD4 T-cell quantification, in addition to the assessment of B-cell subsets and clinical parameters of disease. These updated criteria enable the exclusion of patients with combined immunodeficiency (CID) on the basis of demonstration of severe reductions in naive CD4 T cells. It was suggested that reduced proportions of naive CD4 T cells (<10% CD4 T cells) rather than decreased CD4 counts (<200 cells/μl) are more sensitive in the definition of CID, further highlighting the validity of simultaneously quantifying absolute and relative numbers in patient diagnostics and prognostication protocols. Utilizing the updated criteria, the authors redefined previously published patients on the basis of the updated criteria. Here, 2% patients previously defined as CVID were redefined as CID (122–124). Application of these updated criteria to our CVID group would redefine five (16%) patients as CID. The distinction between CVID and CID is extremely important, as a higher incidence of pneumonia, lymphoma, granulomas, autoimmunity, and enteropathy (2, 5, 123), in addition to lower B-cell and naive CD4 T-cell numbers, accounts for the lower 5-year survival rate in CID patients (123). Thus, unlike PAD patients where IgRT is sufficient for disease management, some patients with CID will require life-saving stem cell transplantation. Hence, the utilization of our panel would aid in the identification and correct classification of patients who progress to CID, as well as PAD patients at risk of developing NICs. We do realize that the differences in cellular immunophenotypes observed in PAD patients with and without NIC will need to be verified in independent international PAD cohorts. Ideally, these markers should be analyzed longitudinally in early-diagnosed patients without NIC to validate if abnormalities will predict disease progression and development of NIC. Therefore, the immunophenotypic defects outlined here could guide genomic analysis of patients, to enable precise diagnosis and predictive prognostic information, as well as guide targeted patient treatment with the potential to limit diagnostic delay, as well as reduce the incidence of early mortality and high morbidity associated with NICs in these patients.

## Data Availability Statement

All datasets generated for this study are included in the article/Supplementary Material.

## Ethics Statement

The studies involving human participants were reviewed and approved by Ethics committee Alfred Health. The patients/participants provided their written informed consent to participate in this study.

## Author Contributions

MZ, RO'H, and JB conceptualized the study and designed the experiments. EE, JB, PA, RS, PC, JC, FH-L, and MZ collected and interpreted the data. EE and PA performed the experiments. EE and MZ wrote the manuscript. All authors critically read, commented on, and approved the final version of the manuscript.

### Conflict of Interest

The authors declare that the research was conducted in the absence of any commercial or financial relationships that could be construed as a potential conflict of interest.

## Abbreviations

CT: cortisone
CVID: common variable immunodeficiency
HGG: hypogammaglobulinemia
IGSCD: IgG subclass deficiency
IgRT: immunoglobulin replacement therapy
MS: multiple sclerosis
PBS: phosphate-buffered saline
RA: rheumatoid arthritis
SLE: systemic lupus erythematosus
SpAD: specific polysaccharide antibody deficiency
XLA: X-linked agammaglobulinemia.

## References

[1] Gathmann B Grimbacher B Beaute J Dudoit Y Mahlaoui N Fischer A. The European internet-based patient and research database for primary immunodeficiencies: results 2006-2008. Clin Exp Immunol. (2009) 157 (Suppl 1):3–11. 10.1111/j.1365-2249.2009.03954.x 19630863 PMC2715433

[2] Picard C Bobby Gaspar H Al-Herz W Bousfiha A Casanova JL Chatila T. International union of immunological societies: 2017 primary immunodeficiency diseases committee report on inborn errors of immunity. J Clin Immunol. (2018) 38:96–128. 10.1007/s10875-017-0464-9 29226302 PMC5742601

[3] Durandy A Kracker S Fischer A. Primary antibody deficiencies. Nat Rev Immunol. (2013) 13:519–33. 10.1038/nri3466 23765059

[4] Lucas M Lee M Lortan J Lopez-Granados E Misbah S Chapel H. Infection outcomes in patients with common variable immunodeficiency disorders: relationship to immunoglobulin therapy over 22 years. J Allergy Clin Immunol. (2010) 125:1354–60.e1354. 10.1016/j.jaci.2010.02.040 20471071

[5] Bousfiha A Jeddane L Picard C Ailal F Bobby Gaspar H Al-Herz W. The 2017 IUIS phenotypic classification for primary immunodeficiencies. J Clin Immunol. (2018) 38:129–43. 10.1007/s10875-017-0465-8 29226301 PMC5742599

[6] Bruton OC. Agammaglobulinemia. Pediatrics. (1952) 9:722–8. 14929630

[7] Tsukada S Saffran DC Rawlings DJ Parolini O Allen RC Klisak I. Deficient expression of a B cell cytoplasmic tyrosine kinase in human X-linked agammaglobulinemia. Cell. (1993) 72:279–90. 10.1016/0092-8674(93)90667-F 8425221

[8] Vetrie D Vorechovsky I Sideras P Holland J Davies A Flinter F. The gene involved in X-linked agammaglobulinaemia is a member of the src family of protein-tyrosine kinases. Nature. (1993) 361:226–33. 10.1038/361226a0 8380905

[9] Dobbs AK Yang T Farmer D Kager L Parolini O Conley ME. Cutting edge: a hypomorphic mutation in Igβ (CD79b) in a patient with immunodeficiency and a leaky defect in B cell development. J Immunol. (2007) 179:2055–9. 10.4049/jimmunol.179.4.2055 17675462

[10] Ferrari S Lougaris V Caraffi S Zuntini R Yang J Soresina A. Mutations of the Igβ gene cause agammaglobulinemia in man. J Exp Med. (2007) 204:2047–51. 10.1084/jem.20070264 17709424 PMC2118692

[11] Minegishi Y Coustan-Smith E Rapalus L Ersoy F Campana D Conley ME. Mutations in Igα (CD79a) result in a complete block in B-cell development. J Clin Invest. (1999) 104:1115–21. 10.1172/JCI7696 10525050 PMC408581

[12] Minegishi Y Coustan-Smith E Wang YH Cooper MD Campana D Conley ME. Mutations in the human lambda5/14.1 gene result in B cell deficiency and agammaglobulinemia. J Exp Med. (1998) 187:71–7. 10.1084/jem.187.1.71 9419212 PMC2199185

[13] Minegishi Y Rohrer J Coustan-Smith E Lederman HM Pappu R Campana D. An essential role for BLNK in human B cell development. Science. (1999) 286:1954–7. 10583958 10.1126/science.286.5446.1954

[14] Yel L Minegishi Y Coustan-Smith E Buckley RH Trubel H Pachman LM. Mutations in the mu heavy-chain gene in patients with agammaglobulinemia. N Engl J Med. (1996) 335:1486–93. 10.1056/NEJM199611143352003 8890099

[15] van Zelm MC Geertsema C Nieuwenhuis N de Ridder D Conley ME Schiff C. Gross deletions involving IGHM, BTK, or Artemis: a model for genomic lesions mediated by transposable elements. Am J Hum Genet. (2008) 82:320–32. 10.1016/j.ajhg.2007.10.011 18252213 PMC2427306

[16] van Zelm MC Reisli I van der Burg M Castano D van Noesel CJ van Tol MJ. An antibody-deficiency syndrome due to mutations in the CD19 gene. N Engl J Med. (2006) 354:1901–12. 10.1056/NEJMoa051568 16672701

[17] van Zelm MC Smet J Adams B Mascart F Schandene L Janssen F. CD81 gene defect in humans disrupts CD19 complex formation and leads to antibody deficiency. J Clin Invest. (2010) 120:1265–74. 10.1172/JCI39748 20237408 PMC2846042

[18] Thiel J Kimmig L Salzer U Grudzien M Lebrecht D Hagena T. Genetic CD21 deficiency is associated with hypogammaglobulinemia. J Allergy Clin Immunol. (2012) 129:801–10.e806. 10.1016/j.jaci.2011.09.027 22035880

[19] Angulo I Vadas O Garcon F Banham-Hall E Plagnol V Leahy TR. Phosphoinositide 3-kinase delta gene mutation predisposes to respiratory infection and airway damage. Science. (2013) 342:866–71. 10.1126/science.1243292 24136356 PMC3930011

[20] Lucas CL Kuehn HS Zhao F Niemela JE Deenick EK Palendira U. Dominant-activating germline mutations in the gene encoding the PI(3)K catalytic subunit p110delta result in T cell senescence and human immunodeficiency. Nat Immunol. (2014) 15:88–97. 10.1038/ni.2771 24165795 PMC4209962

[21] Kuijpers TW Bende RJ Baars PA Grummels A Derks IA Dolman KM. CD20 deficiency in humans results in impaired T cell-independent antibody responses. J Clin Invest. (2010) 120:214–22. 10.1172/JCI40231 20038800 PMC2798692

[22] Tuijnenburg P Lango Allen H Burns SO Greene D Jansen MH Staples E. Loss-of-function nuclear factor kappaB subunit 1 (NFKB1) variants are the most common monogenic cause of common variable immunodeficiency in Europeans. J Allergy Clin Immunol. (2018) 142:1285–96. 10.1016/j.jaci.2018.01.039 29477724 PMC6148345

[23] Warnatz K Bossaller L Salzer U Skrabl-Baumgartner A Schwinger W van der Burg M. Human ICOS deficiency abrogates the germinal center reaction and provides a monogenic model for common variable immunodeficiency. Blood. (2006) 107:3045–52. 10.1182/blood-2005-07-2955 16384931

[24] Lee CE Fulcher DA Whittle B Chand R Fewings N Field M. Autosomal-dominant B-cell deficiency with alopecia due to a mutation in NFKB2 that results in nonprocessable p100. Blood. (2014) 124:2964–72. 10.1182/blood-2014-06-578542 25237204 PMC4321335

[25] Shi C Wang F Tong A Zhang XQ Song HM Liu ZY. NFKB2 mutation in common variable immunodeficiency and isolated adrenocorticotropic hormone deficiency: a case report and review of literature. Medicine. (2016) 95:e5081. 10.1097/MD.0000000000005081 27749582 PMC5059085

[26] Lucas CL Zhang Y Venida A Wang Y Hughes J McElwee J. Heterozygous splice mutation in PIK3R1 causes human immunodeficiency with lymphoproliferation due to dominant activation of PI3K. J Exp Med. (2014) 211:2537–47. 10.1084/jem.20141759 25488983 PMC4267241

[27] van Zelm MC Bartol SJ Driessen GJ Mascart F Reisli I Franco JL. Human CD19 and CD40L deficiencies impair antibody selection and differentially affect somatic hypermutation. J Allergy Clin Immunol. (2014) 134:135–44. 10.1016/j.jaci.2013.11.015 24418477

[28] Maffucci P Filion CA Boisson B Itan Y Shang L Casanova JL. Genetic diagnosis using whole exome sequencing in common variable immunodeficiency. Front Immunol. (2016) 7:220. 10.3389/fimmu.2016.00220 27379089 PMC4903998

[29] Bogaert DJ Dullaers M Lambrecht BN Vermaelen KY De Baere E Haerynck F. Genes associated with common variable immunodeficiency: one diagnosis to rule them all? J Med Genet. (2016) 53:575–90. 10.1136/jmedgenet-2015-103690 27250108

[30] de Valles-Ibanez G Esteve-Sole A Piquer M Gonzalez-Navarro EA Hernandez-Rodriguez J Laayouni H Evaluating the genetics of common variable immunodeficiency: monogenetic model and beyond. Front Immunol. (2018) 9:636 10.3389/fimmu.2018.00636 29867916 PMC5960686

[31] Ameratunga R Brewerton M Slade C Jordan A Gillis D Steele R. Comparison of diagnostic criteria for common variable immunodeficiency disorder. Front Immunol. (2014) 5:415. 10.3389/fimmu.2014.00415 25309532 PMC4164032

[32] Boileau J Mouillot G Gerard L Carmagnat M Rabian C Oksenhendler E. Autoimmunity in common variable immunodeficiency: correlation with lymphocyte phenotype in the French DEFI study. J Autoimmun. (2011) 36:25–32. 10.1016/j.jaut.2010.10.002 21075598

[33] ImmunodeficienciesESF (2017). New Clinical Diagnosis Criteria for the ESID Registry. Geneva Available online at: https://esid.org/Working-Parties/Registry/Diagnosis-criteria

[34] Conley ME Notarangelo LD Etzioni A. Diagnostic criteria for primary immunodeficiencies. Representing PAGID (Pan-American Group for Immunodeficiency) and ESID (European Society for Immunodeficiencies). Clin Immunol. (1999) 93:190–7. 10600329 10.1006/clim.1999.4799

[35] Bonilla FA Barlan I Chapel H Costa-Carvalho BT Cunningham-Rundles C de la Morena MT. International Consensus Document (ICON): common variable immunodeficiency disorders. J Allergy Clin Immunol Pract. (2016) 4:38–59. 10.1016/j.jaip.2015.07.025 26563668 PMC4869529

[36] Driessen GJ Dalm VA van Hagen PM Grashoff HA Hartwig NG van Rossum AM. Common variable immunodeficiency and idiopathic primary hypogammaglobulinemia: two different conditions within the same disease spectrum. Haematologica. (2013) 98:1617–23. 10.3324/haematol.2013.085076 23753020 PMC3789468

[37] Romberg N Le Coz C Glauzy S Schickel JN Trofa M Nolan BE. Patients with common variable immunodeficiency with autoimmune cytopenias exhibit hyperplastic yet inefficient germinal center responses. J Allergy Clin Immunol. (2019) 143:258–65. 10.1016/j.jaci.2018.06.012 29935219 PMC6400323

[38] Slade CA Bosco JJ Binh Giang T Kruse E Stirling RG Cameron PU. Delayed diagnosis and complications of predominantly antibody deficiencies in a cohort of australian adults. Front Immunol. (2018) 9:694. 10.3389/fimmu.2018.00694 29867917 PMC5960671

[39] Wang N Hammarstrom L. IgA deficiency: what is new? Curr Opin Allergy Clin Immunol. (2012) 12:602–8. 10.1097/ACI.0b013e3283594219 23026772

[40] Jolles S. The variable in common variable immunodeficiency: a disease of complex phenotypes. J Allergy Clin Immunol Pract. (2013) 1:545–6. 10.1016/j.jaip.2013.09.015 24565700

[41] Yazdani R Azizi G Abolhassani H Aghamohammadi A. Selective IgA deficiency: epidemiology, pathogenesis, clinical phenotype, diagnosis, prognosis and management. Scand J Immunol. (2017) 85:3–12. 10.1111/sji.12499 27763681

[42] Chapel H Lucas M Patel S Lee M Cunningham-Rundles C Resnick E. Confirmation and improvement of criteria for clinical phenotyping in common variable immunodeficiency disorders in replicate cohorts. J Allergy Clin Immunol. (2012) 130:1197–8.e1199. 10.1016/j.jaci.2012.05.046 22819511

[43] Cunningham-Rundles C. The many faces of common variable immunodeficiency. Hematology Am Soc Hematol Educ Program. (2012) 2012:301–5. 10.1182/asheducation.V2012.1.301.3798316 23233596 PMC4066657

[44] Resnick ES Moshier EL Godbold JH Cunningham-Rundles C. Morbidity and mortality in common variable immune deficiency over 4 decades. Blood. (2012) 119:1650–7. 10.1182/blood-2011-09-377945 22180439 PMC3286343

[45] Odnoletkova I Kindle G Quinti I Grimbacher B Knerr V Gathmann B. The burden of common variable immunodeficiency disorders: a retrospective analysis of the European Society for Immunodeficiency (ESID) registry data. Orphanet J Rare Dis. (2018) 13:201. 10.1186/s13023-018-0941-0 30419968 PMC6233554

[46] Warnatz K Wehr C Drager R Schmidt S Eibel H Schlesier M. Expansion of CD19(hi)CD21(lo/neg) B cells in common variable immunodeficiency (CVID) patients with autoimmune cytopenia. Immunobiology. (2002) 206:502–13. 10.1078/0171-2985-00198 12607725

[47] Wehr C Kivioja T Schmitt C Ferry B Witte T Eren E. The EUROclass trial: defining subgroups in common variable immunodeficiency. Blood. (2008) 111:77–85. 10.1182/blood-2007-06-091744 17898316

[48] Blanco E Perez-Andres M Arriba-Mendez S Serrano C Criado I Del Pino-Molina L Defects in memory B-cell and plasma cell subsets expressing different immunoglobulin-subclasses in patients with CVID and immunoglobulin subclass deficiencies. J Allergy Clin Immunol. (2019) 144:809–24. 10.1016/j.jaci.2019.02.017 30826363

[49] Driessen GJ van Zelm MC van Hagen PM Hartwig NG Trip M Warris A. B-cell replication history and somatic hypermutation status identify distinct pathophysiologic backgrounds in common variable immunodeficiency. Blood. (2011) 118:6814–23. 10.1182/blood-2011-06-361881 22042693

[50] Warnatz K Schlesier M. Flowcytometric phenotyping of common variable immunodeficiency. Cytometry B Clin Cytom. (2008) 74:261–71. 10.1002/cyto.b.20432 18561200

[51] Ebbo M Gerard L Carpentier S Vely F Cypowyj S Farnarier C. Low Circulating natural killer cell counts are associated with severe disease in patients with common variable immunodeficiency. EBioMed. (2016) 6:222–30. 10.1016/j.ebiom.2016.02.025 27211564 PMC4856746

[52] Aspalter RM Sewell WA Dolman K Farrant J Webster AD. Deficiency in circulating natural killer (NK) cell subsets in common variable immunodeficiency and X-linked agammaglobulinaemia. Clin Exp Immunol. (2000) 121:506–14. 10.1046/j.1365-2249.2000.01317.x 10971518 PMC1905722

[53] Giovannetti A Pierdominici M Mazzetta F Marziali M Renzi C Mileo AM. Unravelling the complexity of T cell abnormalities in common variable immunodeficiency. J Immunol. (2007) 178:3932–43. 10.4049/jimmunol.178.6.3932 17339494

[54] Bateman EA Ayers L Sadler R Lucas M Roberts C Woods A. T cell phenotypes in patients with common variable immunodeficiency disorders: associations with clinical phenotypes in comparison with other groups with recurrent infections. Clin Exp Immunol. (2012) 170:202–11. 10.1111/j.1365-2249.2012.04643.x 23039891 PMC3482367

[55] Coraglia A Galassi N Fernandez Romero DS Juri MC Felippo M Malbran A. Common variable immunodeficiency and circulating TFH. J Immunol Res. (2016) 2016:4951587. 10.1155/2016/4951587 27069935 PMC4812460

[56] Unger S Seidl M van Schouwenburg P Rakhmanov M Bulashevska A Frede N. The TH1 phenotype of follicular helper T cells indicates an IFN-γ-associated immune dysregulation in patients with CD21low common variable immunodeficiency. J Allergy Clin Immunol. (2018) 141:730–40. 10.1016/j.jaci.2017.04.041 28554560

[57] Romberg ND Hsu I Price CC Cunningham-Rundles C Meffre E Expansion of circulating t follicular helper cells in CVID patients with autoimmune cytopenias. J Allergy Clin Immunol. (2014) 133:AB162 10.1016/j.jaci.2013.12.586

[58] Melo KM Carvalho KI Bruno FR Ndhlovu LC Ballan WM Nixon DF. A decreased frequency of regulatory T cells in patients with common variable immunodeficiency. PLoS ONE. (2009) 4:e6269. 10.1371/journal.pone.0006269 19649263 PMC2715881

[59] Arumugakani G Wood PM Carter CR. Frequency of Treg cells is reduced in CVID patients with autoimmunity and splenomegaly and is associated with expanded CD21lo B lymphocytes. J Clin Immunol. (2010) 30:292–300. 19997968 10.1007/s10875-009-9351-3

[60] Barbosa RR Silva SP Silva SL Melo AC Pedro E Barbosa MP. Primary B-cell deficiencies reveal a link between human IL-17-producing CD4 T-cell homeostasis and B-cell differentiation. PLoS ONE. (2011) 6:e22848. 10.1371/journal.pone.0022848 21826211 PMC3149619

[61] Kutukculer N Azarsiz E Aksu G Karaca NE. CD4+CD25+Foxp3+ T regulatory cells, Th1 (CCR5, IL-2, IFN-γ) and Th2 (CCR4, IL-4, Il-13) type chemokine receptors and intracellular cytokines in children with common variable immunodeficiency. Int J Immunopathol Pharmacol. (2016) 29:241–51. 10.1177/0394632015617064 26684629 PMC5806726

[62] Kalina T Flores-Montero J van der Velden VH Martin-Ayuso M Bottcher S Ritgen M. EuroFlow standardization of flow cytometer instrument settings and immunophenotyping protocols. Leukemia. (2012) 26:1986–2010. 10.1038/leu.2012.122 22948490 PMC3437409

[63] van der Burg M Kalina T Perez-Andres M Vlkova M Lopez-Granados E Blanco E. The euroFlow PID orientation tube for flow cytometric diagnostic screening of primary immunodeficiencies of the lymphoid system. Front Immunol. (2019) 10:246. 10.3389/fimmu.2019.00246 30886612 PMC6410673

[64] van Dongen JJM van der Burg M Kalina T Perez-Andres M Mejstrikova E Vlkova M. EuroFlow-based flowcytometric diagnostic screening and classification of primary immunodeficiencies of the lymphoid system. Front Immunol. 10:1271. 10.3389/fimmu.2019.01271 31263462 PMC6585843

[65] van der Velden VH Flores-Montero J Perez-Andres M Martin-Ayuso M Crespo O Blanco E. *Optimization and testing of dried antibody* tube: the EuroFlow LST and PIDOT tubes as examples. J Immunol Methods. (2017) S0022-1759(17)30095-9. 10.1016/j.jim.2017.03.011 28341440

[66] Heeringa JJ Rijvers L Arends NJ Driessen GJ Pasmans SG van Dongen JJM. IgE-expressing memory B cells and plasmablasts are increased in blood of children with asthma, food allergy, and atopic dermatitis. Allergy. (2018) 73:1331–6. 10.1111/all.13421 29380876

[67] Liu W Putnam AL Xu-Yu Z Szot GL Lee MR Zhu S. CD127 expression inversely correlates with FoxP3 and suppressive function of human CD4+ T reg cells. J Exp Med. (2006) 203:1701–11. 10.1084/jem.20060772 16818678 PMC2118339

[68] Seddiki N Santner-Nanan B Martinson J Zaunders J Sasson S Landay A. Expression of interleukin (IL)-2 and IL-7 receptors discriminates between human regulatory and activated T cells. J Exp Med. (2006) 203:1693–700. 10.1084/jem.20060468 16818676 PMC2118333

[69] Annunziato F Cosmi L Liotta F Maggi E Romagnani S. The phenotype of human Th17 cells and their precursors, the cytokines that mediate their differentiation and the role of Th17 cells in inflammation. Int Immunol. (2008) 20:1361–8. 10.1093/intimm/dxn106 18820263

[70] Bonecchi R Bianchi G Bordignon PP D'Ambrosio D Lang R Borsatti A. Differential expression of chemokine receptors and chemotactic responsiveness of type 1 T helper cells (Th1s) and Th2s. J Exp Med. (1998) 187:129–34. 10.1084/jem.187.1.129 9419219 PMC2199181

[71] Breitfeld D Ohl L Kremmer E Ellwart J Sallusto F Lipp M. Follicular B helper T cells express CXC chemokine receptor 5, localize to B cell follicles, and support immunoglobulin production. J Exp Med. (2000) 192:1545–52. 10.1084/jem.192.11.1545 11104797 PMC2193094

[72] Schaerli P Willimann K Lang AB Lipp M Loetscher P Moser B. CXC chemokine receptor 5 expression defines follicular homing T cells with B cell helper function. J Exp Med. (2000) 192:1553–62. 10.1084/jem.192.11.1553 11104798 PMC2193097

[73] Ma CS Wong N Rao G Avery DT Torpy J Hambridge T. Monogenic mutations differentially affect the quantity and quality of T follicular helper cells in patients with human primary immunodeficiencies. J Allergy Clin Immunol. (2015) 136.e1001. 10.1016/j.jaci.2015.05.036 26162572 PMC5042203

[74] Blanco E Perez-Andres M Arriba-Mendez S Contreras-Sanfeliciano T Criado I Pelak O. Age-associated distribution of normal B-cell and plasma cell subsets in peripheral blood. J Allergy Clin Immunol. (2018) 141:2208–19.e2216. 29505809 10.1016/j.jaci.2018.02.017

[75] Flores-Montero J Sanoja-Flores L Paiva B Puig N Garcia-Sanchez O Bottcher S. Next generation flow for highly sensitive and standardized detection of minimal residual disease in multiple myeloma. Leukemia. (2017) 31:2094–103. 10.1038/leu.2017.29 28104919 PMC5629369

[76] Theunissen P Mejstrikova E Sedek L van der Sluijs-Gelling AJ Gaipa G Bartels M. Standardized flow cytometry for highly sensitive MRD measurements in B-cell acute lymphoblastic leukemia. Blood. (2017) 129:347–57. 10.1182/blood-2016-07-726307 27903527 PMC5291958

[77] Verstegen RHJ Aui PM Watson E De Jong S Bartol SJW Bosco JJ. Quantification of T-cell and B-cell replication history in aging, immunodeficiency, and newborn screening. Front Immunol. (2019) 10:2084. 10.3389/fimmu.2019.02084 31543882 PMC6730487

[78] van Zelm MC Pumar M Shuttleworth P Aui PM Smart JM Grigg A. Functional antibody responses following allogeneic stem cell transplantation for TP53 mutant pre-B-ALL in a patient with X-linked agammaglobulinemia. Front Immunol. (2019) 10:895. 10.3389/fimmu.2019.00895 31105705 PMC6498405

[79] Warnatz K Denz A Drager R Braun M Groth C Wolff-Vorbeck G. Severe deficiency of switched memory B cells (CD27(+)IgM(-)IgD(-)) in subgroups of patients with common variable immunodeficiency: a new approach to classify a heterogeneous disease. Blood. (2002) 99:1544–51. 10.1182/blood.V99.5.1544 11861266

[80] van den Heuvel D Jansen MA Dik WA Bouallouch-Charif H Zhao D van Kester KA. Cytomegalovirus- and epstein-barr virus-induced T-cell expansions in young children do not impair naive T-cell populations or vaccination responses: the generation R study. J Infect Dis. (2016) 213:233–42. 10.1093/infdis/jiv369 26142434

[81] Romberg N Chamberlain N Saadoun D Gentile M Kinnunen T Ng YS. CVID-associated TACI mutations affect autoreactive B cell selection and activation. J Clin Invest. (2013) 123:4283–93. 10.1172/JCI69854 24051380 PMC3786721

[82] Cunningham-Rundles C. Common variable immune deficiency: dissection of the variable. Immunol Rev. (2019) 287:145–61. 10.1111/imr.12728 30565247 PMC6435035

[83] Chapel H Lucas M Lee M Bjorkander J Webster D Grimbacher B. Common variable immunodeficiency disorders: division into distinct clinical phenotypes. Blood. (2008) 112:277–86. 10.1182/blood-2007-11-124545 18319398

[84] Agarwal S Cunningham-Rundles C. Autoimmunity in common variable immunodeficiency. Curr Allergy Asthma Rep. (2009) 9:347–52. 10.1007/s11882-009-0051-0 19671377 PMC2919211

[85] Warnatz K Voll RE. Pathogenesis of autoimmunity in common variable immunodeficiency. Front Immunol. (2012) 3:210. 10.3389/fimmu.2012.00210 22826712 PMC3399211

[86] Gathmann B Mahlaoui N Ceredih GL Oksenhendler E Warnatz K European Society for Immunodeficiencies Registry Working P. Clinical picture and treatment of 2212 patients with common variable immunodeficiency. J Allergy Clin Immunol. (2014) 134:116–26. 10.1016/j.jaci.2013.12.1077 24582312

[87] Stuchly J Kanderova V Vlkova M Hermanova I Slamova L Pelak O Common variable immunodeficiency patients with a phenotypic profile of immunosenescence present with thrombocytopenia. Sci Rep. (2017) 7:39710 10.1038/srep39710 28054583 PMC5214528

[88] Boes M. Role of natural and immune IgM antibodies in immune responses. Mol Immunol. (2000) 37:1141–9. 10.1016/s0161-5890(01)00025-6 11451419

[89] Chen Y Park YB Patel E Silverman GJ. IgM antibodies to apoptosis-associated determinants recruit C1q and enhance dendritic cell phagocytosis of apoptotic cells. J Immunol. (2009) 182:6031–43. 10.4049/jimmunol.0804191 19414754 PMC4428684

[90] Timmermans WM van Laar JA van der Houwen TB Kamphuis LS Bartol SJ Lam KH. B-cell dysregulation in crohn's disease is partially restored with infliximab therapy. PLoS ONE. (2016) 11:e0160103. 10.1371/journal.pone.0160103 27468085 PMC4965034

[91] Saadoun D Terrier B Bannock J Vazquez T Massad C Kang I. Expansion of autoreactive unresponsive CD21-/low B cells in Sjogren's syndrome-associated lymphoproliferation. Arthritis Rheum. (2013) 65:1085–96. 10.1002/art.37828 23279883 PMC4479193

[92] van den Heuvel D Driessen GJ Berkowska MA van der Burg M Langerak AW Zhao D. Persistent subclinical immune defects in HIV-1-infected children treated with antiretroviral therapy. AIDS. (2015) 29:1745–56. 10.1097/QAD.0000000000000765 26372381

[93] McComish J Mundy J Sullivan T Proudman SM Hissaria P. Changes in peripheral blood B cell subsets at diagnosis and after treatment with disease-modifying anti-rheumatic drugs in patients with rheumatoid arthritis: correlation with clinical and laboratory parameters. Int J Rheum Dis. (2015) 18:421–32. 10.1111/1756-185X.12325 24589014

[94] Rubtsov AV Rubtsova K Fischer A Meehan RT Gillis JZ Kappler JW. Toll-like receptor 7 (TLR7)-driven accumulation of a novel CD11c(+) B-cell population is important for the development of autoimmunity. Blood. (2011) 118:1305–15. 10.1182/blood-2011-01-331462 21543762 PMC3152497

[95] Wehr C Eibel H Masilamani M Illges H Schlesier M Peter HH. A new CD21low B cell population in the peripheral blood of patients with SLE. Clin Immunol. (2004) 113:161–71. 10.1016/j.clim.2004.05.010 15451473

[96] Claes N Fraussen J Vanheusden M Hellings N Stinissen P Van Wijmeersch B. Age-associated B cells with proinflammatory characteristics are expanded in a proportion of multiple sclerosis patients. J Immunol. (2016) 197:4576–83. 10.4049/jimmunol.1502448 27837111

[97] Fagnoni FF Vescovini R Passeri G Bologna G Pedrazzoni M Lavagetto G. Shortage of circulating naive CD8(+) T cells provides new insights on immunodeficiency in aging. Blood. (2000) 95:2860–8. 10.1182/blood.V95.9.2860.009k35_2860_2868 10779432

[98] Rabin RL Roederer M Maldonado Y Petru A Herzenberg LA Herzenberg LA. Altered representation of naive and memory CD8 T cell subsets in HIV-infected children. J Clin Invest. (1995) 95:2054–60. 10.1172/JCI117891 7738172 PMC295792

[99] Roederer M Dubs JG Anderson MT Raju PA Herzenberg LA Herzenberg LA. CD8 naive T cell counts decrease progressively in HIV-infected adults. J Clin Invest. (1995) 95:2061–6. 10.1172/JCI117892 7738173 PMC295794

[100] Anyimadu H Pingili C Sivapalan V Hirsch-Moverman Y Mannheimer S. The impact of absolute CD4 count and percentage discordance on pneumocystis jirovecii pneumonia prophylaxis in HIV-infected patients. J Int Assoc Provid AIDS Care. (2018) 17:2325958218759199. 10.1177/2325958218759199 29534652 PMC6748489

[101] Long SA Buckner JH. CD4+FOXP3+ T regulatory cells in human autoimmunity: more than a numbers game. J Immunol. (2011) 187:2061–6. 10.4049/jimmunol.1003224 21856944 PMC3160735

[102] Brusko TM Wasserfall CH Clare-Salzler MJ Schatz DA Atkinson MA. Functional defects and the influence of age on the frequency of CD4+ CD25+ T-cells in type 1 diabetes. Diabetes. (2005) 54:1407–14. 10.2337/diabetes.54.5.1407 15855327

[103] Putnam AL Vendrame F Dotta F Gottlieb PA. CD4+CD25high regulatory T cells in human autoimmune diabetes. J Autoimmun. (2005) 24:55–62. 10.1016/j.jaut.2004.11.004 15725577

[104] Astier AL Meiffren G Freeman S Hafler DA. Alterations in CD46-mediated Tr1 regulatory T cells in patients with multiple sclerosis. J Clin Invest. (2006) 116:3252–7. 10.1172/JCI29251 17099776 PMC1635165

[105] Huan J Culbertson N Spencer L Bartholomew R Burrows GG Chou YK. Decreased FOXP3 levels in multiple sclerosis patients. J Neurosci Res. (2005) 81:45–52. 10.1002/jnr.20522 15952173

[106] Zhang B Zhang X Tang F Zhu L Liu Y. Reduction of forkhead box P3 levels in CD4+CD25high T cells in patients with new-onset systemic lupus erythematosus. Clin Exp Immunol. (2008) 153:182–7. 10.1111/j.1365-2249.2008.03686.x 18505426 PMC2492897

[107] Peck A Mellins ED. Precarious balance: Th17 cells in host defense. Infect Immun. (2010) 78:32–8. 10.1128/IAI.00929-09 19901061 PMC2798221

[108] Crum-Cianflone N Weekes J Bavaro M. Recurrent community-associated methicillin-resistant Staphylococcus aureus infections among HIV-infected persons: incidence and risk factors. AIDS Patient Care STDS. (2009) 23:499–502. 10.1089/apc.2008.0240 19530952 PMC2732573

[109] Hirschtick RE Glassroth J Jordan MC Wilcosky TC Wallace JM Kvale PA. Bacterial pneumonia in persons infected with the human immunodeficiency virus. *Pulmonary* c*omplications of HIV* infection study group. N Engl J Med. (1995) 333:845–51. 7651475 10.1056/NEJM199509283331305

[110] Minegishi Y Saito M Nagasawa M Takada H Hara T Tsuchiya S. Molecular explanation for the contradiction between systemic Th17 defect and localized bacterial infection in hyper-IgE syndrome. J Exp Med. (2009) 206:1291–301. 10.1084/jem.20082767 19487419 PMC2715068

[111] Milner JD Brenchley JM Laurence A Freeman AF Hill BJ Elias KM. Impaired T(H)17 cell differentiation in subjects with autosomal dominant hyper-IgE syndrome. Nature. (2008) 452:773–6. 10.1038/nature06764 18337720 PMC2864108

[112] Paulson ML Freeman AF Holland SM. Hyper IgE syndrome: an update on clinical aspects and the role of signal transducer and activator of transcription 3. Curr Opin Allergy Clin Immunol. (2008) 8:527–33. 10.1097/ACI.0b013e3283184210 18978467

[113] Xie J Cui D Liu Y Jin J Tong H Wang L. Changes in follicular helper T cells in idiopathic thrombocytopenic purpura patients. Int J Biol Sci. (2015) 11:220–9. 10.7150/ijbs.10178 25561904 PMC4279097

[114] Gensous N Charrier M Duluc D Contin-Bordes C Truchetet ME Lazaro E. T follicular helper cells in autoimmune disorders. Front Immunol. (2018) 9:1637. 10.3389/fimmu.2018.01637 30065726 PMC6056609

[115] Romme Christensen J Bornsen L Ratzer R Piehl F Khademi M Olsson T Systemic inflammation in progressive multiple sclerosis involves follicular T-helper, Th17- and activated B-cells and correlates with progression. PLoS ONE. (2013) 8:e57820 10.1371/journal.pone.0057820 23469245 PMC3585852

[116] Rodriguez-Perea AL Arcia ED Rueda CM Velilla PA. Phenotypical characterization of regulatory T cells in humans and rodents. Clin Exp Immunol. (2016) 185:281–91. 10.1111/cei.12804 27124481 PMC4991523

[117] Annunziato F Cosmi L Santarlasci V Maggi L Liotta F Mazzinghi B. Phenotypic and functional features of human Th17 cells. J Exp Med. (2007) 204:1849–61. 10.1084/jem.20070663 17635957 PMC2118657

[118] Sallusto F Lanzavecchia A. Heterogeneity of CD4+ memory T cells: functional modules for tailored immunity. Eur J Immunol. (2009) 39:2076–82. 10.1002/eji.200939722 19672903

[119] Kilpatrick RD Rickabaugh T Hultin LE Hultin P Hausner MA Detels R. Homeostasis of the naive CD4+ T cell compartment during aging. J Immunol. (2008) 180:1499–507. 10.4049/jimmunol.180.3.1499 18209045 PMC2940825

[120] Czesnikiewicz-Guzik M Lee WW Cui D Hiruma Y Lamar DL Yang ZZ. T cell subset-specific susceptibility to aging. Clin Immunol. (2008) 127:107–18. 10.1016/j.clim.2007.12.002 18222733 PMC2435295

[121] Wertheimer AM Bennett MS Park B Uhrlaub JL Martinez C Pulko V. Aging and cytomegalovirus infection differentially and jointly affect distinct circulating T cell subsets in humans. J Immunol. (2014) 192:2143–55. 10.4049/jimmunol.1301721 24501199 PMC3989163

[122] von Spee-Mayer C Koemm V Wehr C Goldacker S Kindle G Bulashevska A Evaluating laboratory criteria for combined immunodeficiency in adult patients diagnosed with common variable immunodeficiency. Clin Immunol. (2019) 203:59–62. 10.1016/j.clim.2019.04.001 31004792

[123] Bertinchamp R Gerard L Boutboul D Malphettes M Fieschi C Oksenhendler E. Exclusion of Patients with a Severe T-cell defect improves the definition of common variable immunodeficiency. J Allergy Clin Immunol Pract. (2016) 4:1147–57. 10.1016/j.jaip.2016.07.002 27522107

[124] Chapel H. Common variable immunodeficiency disorders (CVID) - diagnoses of exclusion, especially combined immune defects. J Allergy Clin Immunol Pract. (2016) 4:1158–9. 10.1016/j.jaip.2016.09.006 27836061

