# Supplementary material for: Predominantly Antibody-Deficient Patients With Non-infectious Complications Have Reduced Naive B, Treg, Th17, and Tfh17 Cells
Source: Front Immunol. 2019 Nov 15;10:2593. doi: 10.3389/fimmu.2019.02593 (PMC6873234; doi:10.3389/fimmu.2019.02593)
Supplement: Supplementary file 1 [file Data_Sheet_1.pdf]

Supplementary Materials

Supplementary Table 1. Composition of the antibody panels

tube	fluorochrome										
	**BV421**	**BV510**	**BV605**	**BV711**	**FITC/ BB515**	**PerCP-Cy5.5**	**PE**	**PE-Dazzle**	**PC7 / PE-Cy7**	**APC**	**APC-H7 / APC-Ax750**
1. TruCount	-	-	-	-	CD3	CD45	CD16 + CD56	-	CD4	CD19	CD8A
2. B-cell	CD27	IgM	CD38	CD21	IgE + IgA	IgD	IgG + IgA	-	CD19	CD5	CD24
3. T-effector	CD27	CD4	CD45RA	CD3	CD45RO	CD28	CD31	CCR7	TCRγδ	HLA-DR	CD8A
4. Th subset	CD25	CD4	CD45RA	CD3	CXCR5	CCR6	CXCR3	CCR7	CCR4	CD127	CD8A

Supplementary Table 2. Antibody list

Marker	Fluorochrome	Clone	Source	Cat. number	volume/ test (in µl)	tube(s)
CD3	FITC	UCHT1	BD Biosciences	555332	3	1
CD3	BV711	UCHT1	BD Biosciences	563725	2.5	3, 4
CD4	PC7	SFCI12T4D11	Beckman Coulter	6607101	0.2	1
CD4	BV510	RPA-T4	Biolegend	300546	1.5	3, 4
CD5	APC	UCHT2	Biolegend	300612	0.2	2
CD8A	APC-H7	SK1	BD Biosciences	560179	4	1, 3, 4
CD16	PE	B73.1	Biolegend	360704	0.2	1
CD19	APC	SJ25C1	Biolegend	363006	0.4	1
CD19	PE-CY7	SJ25C1	BD Biosciences	557835	5	2
CD21	BV711	B-ly4	BD Biosciences	563163	5	2
CD24	APC-Alexa750	ALB9	Beckman Coulter	B10738	2.5	2
CD25	BV421	BC96	Biolegend	302630	2.5	4
CD27	BV421	M-T271	BD Biosciences	562513	1	2, 3
CD28	PerCP-Cy5.5	CD28.2	Biolegend	302922	5	3
CD31	PE	WM59	BD Biosciences	555446	5	3
CD38	BV605	HB7	BD Biosciences	562665	0.2	2
CD45	PerCP-Cy5.5	2D1	BD Biosciences	340953	2	1
CD45RA	BV605	HI100	Biolegend	304134	0.2	3, 4
CD45RO	FITC	UCHL1	Biolegend	304204	5	3
CD56	PE	B159	BD Biosciences	555516	5	1
CD127	APC	A019D5	Biolegend	351316	5	4
CCR4	PE-CY7	L291H4	Biolegend	359410	5	4
CCR6	PerCP-CY5.5	G034E3	Biolegend	353406	2.5	4
CCR7	PE-Dazzle	G043H7	Biolegend	353236	2.5	3, 4
*CCR7	PE-CF594	150503	BD Biosciences	562381	5	3, 4
CXCR3	PE	1C6/CXCR3	BD Biosciences	557185	20	4
CXCR5	BB515	RF8B2	BD Biosciences	564624	5	4
HLA-DR	APC	L243	Biolegend	307610	2	3
IgA	VioBright FITC	IS11-8E10	Miltenyi Biotec	130-104-726	0.5	2
IgA	PE	IS11-8E10	Miltenyi Biotec	130-093-128	0.5	2
IgD	PerCP-Cy5.5	IA6-2	Biolegend	348208	1.5	2
IgE	FITC	goat polyclonal	Invitrogen	H15701	1	2
IgG	PE	G18-145	BD Biosciences	555787	5	2
IgM	BV510	MHM-88	Biolegend	314522	1	2
TCRγδ	PC7	IMMU510	Beckman Coulter	B10247	1	3

* alternative reagent

Supplementary Table 3. Definition of leukocyte subsets

n=55	Population name	Phenotype definition
**Tube 1: Trucount**		
1	Granulocytes	$SSC^{high}CD45^{+}$
2	Monocytes	$SSC^{inter}CD45^{+}$
3	Lymphocytes	$SSC^{low}CD45^{+}$
4	∟B cells	$SSC^{low}CD45^{+}CD19^{+}$
5	∟NK cells	$SSC^{low}CD45^{+}CD3^{-}CD16^{+}$ and/or $CD56^{+}$
6	∟T cells	$SSC^{low}CD45^{+}CD3^{+}CD16^{-}$or $CD56^{-}$
	∟$CD4^{+}$ T cells	$SSC^{low}CD45^{+}CD3^{+}CD16^{-}$or $CD56^{-}CD4^{+}CD8^{-}$
	∟$CD8^{+}$ T cells	$SSC^{low}CD45^{+}CD3^{+}CD16^{-}$or $CD56^{-}CD4^{-}CD8^{+}$
**Tube 2: B cells**		
	B cells	$FSC^{low}SSC^{low}CD19^{+}$
7	transitional B cells	$FSC^{low}SSC^{low}CD19^{+}CD38^{hi}CD27^{-}$
8	plasmablasts	$FSC^{low}SSC^{low}CD19^{+}CD38^{hi}CD27^{+}$
9	$CD21^{lo}$ B cells	$FSC^{low}SSC^{low}CD19^{+}CD38^{low}CD21^{lo}$
	CD21+ B cells	$FSC^{low}SSC^{low}CD19^{+}CD38^{low}CD21^{+}$
	∟CD21+CD27-	$FSC^{low}SSC^{low}CD19^{+}CD38^{low}CD21^{+}CD27^{-}$
10	∟ naive B cells	$FSC^{low}SSC^{low}CD19^{+}CD38^{low}CD21^{+}CD27^{-}IgM^{+}IgD^{+/-}$
11	∟pre-naive	$FSC^{low}SSC^{low}CD19^{+}CD38^{low}CD21^{+}CD27^{-}IgM^{+}IgD^{+/-}CD5^{+}$
12	∟naive/mature	$FSC^{low}SSC^{low}CD19^{+}CD38^{low}CD21^{+}CD27^{-}IgM^{+}IgD^{+/-}CD5^{-}$
	∟$CD27^{-}$ Memory	$FSC^{low}SSC^{low}CD19^{+}CD38^{low}CD21^{+}CD27^{-}IgM^{-}IgD^{-}$
13	∟IgG^{+} $CD27^{-}$ memory	$FSC^{low}SSC^{low}CD19^{+}CD38^{low}CD21^{+}CD27^{-}IgM^{-}IgD^{-}IgG^{+}$
14	∟IgA^{+} $CD27^{-}$ memory	$FSC^{low}SSC^{low}CD19^{+}CD38^{low}CD21^{+}CD27^{-}IgM^{-}IgD^{-}IgA^{+}$
	∟IgE^{+} $CD27^{-}$ memory	$FSC^{low}SSC^{low}CD19^{+}CD38^{low}CD21^{+}CD27^{-}IgM^{-}IgD^{-}IgE^{+}$
	∟CD21+CD27+	$FSC^{low}SSC^{low}CD19^{+}CD38^{low}CD21^{+}CD27^{+}$
15	∟natural effector	$FSC^{low}SSC^{low}CD19^{+}CD38^{low}CD21^{+}CD27^{+}IgM^{+}IgD^{+}$
16	∟IgM only	$FSC^{low}SSC^{low}CD19^{+}CD38^{low}CD21^{+}CD27^{+}IgM^{+}IgD^{-}$
17	∟IgG^{+} $CD27^{+}$ memory	$FSC^{low}SSC^{low}CD19^{+}CD38^{low}CD21^{+}CD27^{+}IgM^{-}IgD^{-}IgA^{+}$
18	∟IgA^{+} $CD27^{+}$ memory	$FSC^{low}SSC^{low}CD19^{+}CD38^{low}CD21^{+}CD27^{+}IgM^{-}IgD^{-}IgA^{+}$
	∟IgE^{+} $CD27^{+}$ memory	$FSC^{low}SSC^{low}CD19^{+}CD38^{low}CD21^{+}CD27^{+}IgM^{-}IgD^{-}IgE^{+}$
**Tube 3: T effector**		
	T cells	$FSC^{low}SSC^{low}CD3^{+}$
19	$\gamma\delta^{+}$ T cells	$FSC^{low}SSC^{low}CD3^{+}\gamma\delta^{+}$
	$\gamma\delta^{-}$ T cells	$FSC^{low}SSC^{low}CD3^{+}\gamma\delta^{-}$
20	∟CD4 T cells	$FSC^{low}SSC^{low}CD3^{+}\gamma\delta^{-}CD4^{+}CD8^{-}$
21	∟CD4 Tnaive	$FSC^{low}SSC^{low}CD3^{+}\gamma\delta^{-}CD4^{+}CD8^{-}CCR7^{+}CD45RO^{-}$
22	∟CD4 Tcm	$FSC^{low}SSC^{low}CD3^{+}\gamma\delta^{-}CD4^{+}CD8^{-}CCR7^{+}CD45RO^{+}$
23	∟CD4 TemRO	$FSC^{low}SSC^{low}CD3^{+}\gamma\delta^{-}CD4^{+}CD8^{-}CCR7^{-}CD45RO^{+}$
24	∟CD4 early TemRO	$FSC^{low}SSC^{low}CD3^{+}\gamma\delta^{-}CD4^{+}CD8^{-}CCR7^{-}CD45RO^{+}CD27^{+}CD28^{+}$
25	∟CD4 intermediate TemRO	$FSC^{low}SSC^{low}CD3^{+}\gamma\delta^{-}CD4^{+}CD8^{-}CCR7^{-}CD45RO^{+}CD27^{-}CD28^{+}$
26	∟CD4 late TemRO	$FSC^{low}SSC^{low}CD3^{+}\gamma\delta^{-}CD4^{+}CD8^{-}CCR7^{-}CD45RO^{+}CD27^{-}CD28^{-}$
27	∟CD4 TemRA	$FSC^{low}SSC^{low}CD3^{+}\gamma\delta^{-}CD4^{+}CD8^{-}CCR7^{-}CD45RO^{-}$
28	∟CD4 early TemRA	$FSC^{low}SSC^{low}CD3^{+}\gamma\delta^{-}CD4^{+}CD8^{-}CCR7^{-}CD45RO^{-}CD27^{+}CD28^{+}$
29	∟CD4 intermediate TemRA	$FSC^{low}SSC^{low}CD3^{+}\gamma\delta^{-}CD4^{+}CD8^{-}CCR7^{-}CD45RO^{-}CD27^{-}CD28^{+}$
30	∟CD4 late TemRA	$FSC^{low}SSC^{low}CD3^{+}\gamma\delta^{-}CD4^{+}CD8^{-}CCR7^{-}CD45RO^{-}CD27^{-}CD28^{-}$
31	∟CD8 T cells	$FSC^{low}SSC^{low}CD3^{+}\gamma\delta^{-}CD4^{-}CD8^{+}$
32	∟CD8 Tnaive	$FSC^{low}SSC^{low}CD3^{+}\gamma\delta^{-}CD4^{-}CD8^{+}CCR7^{+}CD45RO^{-}$
33	∟ CD8 Tcm	$FSC^{low}SSC^{low}CD3^{+}\gamma\delta^{-}CD4^{-}CD8^{+}CCR7^{+}CD45RO^{+}$
34	∟ CD8 TemRO	$FSC^{low}SSC^{low}CD3^{+}\gamma\delta^{-+}CD4^{-}CD8^{+}CCR7^{-}CD45RO^{+}$
35	∟CD8 early TemRO	$FSC^{low}SSC^{low}CD3^{+}\gamma\delta^{-}CD4^{-}CD8^{+}CCR7^{-}CD45RO^{+}CD27^{+}CD28^{+}$
36	∟CD8 intermediate TemRO	$FSC^{low}SSC^{low}CD3^{+}\gamma\delta^{-}CD4^{-}CD8^{+}CCR7^{-}CD45RO^{+}CD27^{+}CD28^{-}$
37	∟CD8 late TemRO	$FSC^{low}SSC^{low}CD3^{+}\gamma\delta^{-}CD4^{-}CD8^{+}CCR7^{-}CD45RO^{+}CD27^{-}CD28^{-}$
38	∟CD8 TemRA	$FSC^{low}SSC^{low}CD3^{+}\gamma\delta^{-}CD4^{-}CD8^{-+}CCR7^{-}CD45RO^{-}$
39	∟CD8 early TemRA	$FSC^{low}SSC^{low}CD3^{+}\gamma\delta^{-}CD4^{-}CD8^{-+}CCR7^{-}CD45RO^{-}CD27^{+}CD28^{+}$
40	∟CD8 intermediate TemRA	$FSC^{low}SSC^{low}CD3^{+}\gamma\delta^{-}CD4^{-}CD8^{-+}CCR7^{-}CD45RO^{-}CD27^{+}CD28^{-}$
41	∟CD8 late TemRA	$FSC^{low}SSC^{low}CD3^{+}\gamma\delta^{-}CD4^{-}CD8^{-+}CCR7^{-}CD45RO^{-}CD27^{-}CD28^{-}$
**Tube 4: Th subset**		
	T Cells	$FSC^{low}SSC^{low}CD3^{+}$

	CD8 T Cells	$FSC^{low}SSC^{low}CD3^{+}CD4^{-}CD8^{+}$
42	∟CD8 Tfh Cells	$FSC^{low}SSC^{low}CD3^{+}CD4^{-}CD8^{+}CXCR5^{+}CD45RA^{-}$
	CD4 T Cells	$FSC^{low}SSC^{low}CD3^{+}CD4^{+}CD8^{-}$
43	∟Treg cells	$FSC^{low}SSC^{low}CD3^{+}CD4^{+}CD8^{-}CD25^{+}CD127^{lo/-}$
44	∟naive Treg	$FSC^{low}SSC^{low}CD3^{+}CD4^{+}CD8^{-}CD25^{+}CD127^{lo}CCR4^{+}CD45RA+$
45	∟memory Treg	$FSC^{low}SSC^{low}CD3^{+}CD4^{+}CD8^{-}CD25^{+}CD127^{lo}CCR4^{-}CD45RA-$
46	∟CD4 Tfr	$FSC^{low}SSC^{low}CD3^{+}CD4^{+}CD8^{-}CD25^{+}CD127^{lo}CXCR5^{+}CD45RA^{-}$
	∟Non-Treg cells	$FSC^{low}SSC^{low}CD3^{+}CD4^{+}CD8^{-}CD25^{-}CD127^{lo/hi}$
	∟Th Cells	$FSC^{low}SSC^{low}CD3^{+}CD4^{+}CD8^{-}CD25^{-}CD127^{lo/hi}CXCR5^{-}CD45RA^{-}$
	∟CCR6- Th	$FSC^{low}SSC^{low}CD3^{+}CD4^{+}CD8^{-}CD25^{-}CD127^{lo/hi}CXCR5^{-}CD45RA^{-}CCR6^{-}$
47	∟Th1	$FSC^{low}SSC^{low}CD3^{+}CD4^{+}CD8^{-}CD25^{-}CD127^{lo/hi}CXCR5^{-}CD45RA^{-}CCR6^{-}CCR4^{-}CXCR3^{+}$
48	∟Th2	$FSC^{low}SSC^{low}CD3^{+}CD4^{+}CD8^{-}CD25^{-}CD127^{lo/hi}CXCR5^{-}CD45RA^{-}CCR6^{-}CCR4^{+}CXCR3^{-}$
	∟CCR6+ Th	$FSC^{low}SSC^{low}CD3^{+}CD4^{+}CD8^{-}CD25^{-}CD127^{lo/hi}CXCR5^{-}CD45RA^{-}CCR6^{+}$
49	∟Th17	$FSC^{low}SSC^{low}CD3^{+}CD4^{+}CD8^{-}CD25^{-}CD127^{lo/hi}CXCR5^{-}CD45RA^{-}CCR6^{+}CCR4^{+}CXCR3^{-}$
50	∟Th17.1	$FSC^{low}SSC^{low}CD3^{+}CD4^{+}CD8^{-}CD25^{-}CD127^{lo/hi}CXCR5^{-}CD45RA^{-}CCR6^{+}CCR4^{-}CXCR3^{+}$
51	∟Tfh Cells	$FSC^{low}SSC^{low}CD3^{+}CD4^{+}CD8^{-}CD25^{-}CD127^{lo/hi}CXCR5^{+}CD45RA^{-}$
	∟CCR6- Tfh	$FSC^{low}SSC^{low}CD3^{+}CD4^{+}CD8^{-}CD25^{-}CD127^{lo/hi}CXCR5^{+}CD45RA^{-}CCR6^{-}$
52	∟Tfh1	$FSC^{low}SSC^{low}CD3^{+}CD4^{+}CD8^{-}CD25^{-}CD127^{lo/hi}CXCR5^{+}CD45RA^{-}CCR6^{-}CCR4^{-}CXCR3^{+}$
53	∟Tfh2	$FSC^{low}SSC^{low}CD3^{+}CD4^{+}CD8^{-}CD25^{-}CD127^{lo/hi}CXCR5^{+}CD45RA^{-}CCR6^{-}CCR4^{+}CXCR3^{-}$
	∟CCR6+ Tfh	$FSC^{low}SSC^{low}CD3^{+}CD4^{+}CD8^{-}CD25^{-}CD127^{lo/hi}CXCR5^{+}CD45RA^{-}CCR6^{+}$
54	∟Tfh17	$FSC^{low}SSC^{low}CD3^{+}CD4^{+}CD8^{-}CD25^{-}CD127^{lo/hi}CXCR5^{+}CD45RA^{-}CCR6^{+}CCR4^{+}CXCR3^{-}$
55	∟Tfh17.1	$FSC^{low}SSC^{low}CD3^{+}CD4^{+}CD8^{-}CD25^{-}CD127^{lo/hi}CXCR5^{+}CD45RA^{-}CCR6^{+}CCR4^{-}CXCR3^{+}$

Supplementary Table 4. Flowcytometer set-up

**LSRFortessa X-20**		**LSR-II and LSRFortessa**		**Fluorochromes used in this study**
**355 nm**		-		-
379/28	No LP	-	-	-
525/50	505 LP	-	-	-
740/35	690 LP	-	-	-
**405 nm**		**405 nm**		
450/50	No LP	450/50	No LP	BV421
525/50	505 LP	525/50	505 LP	BV510
-	-	586/15	570 LP	-
610/20	600 LP	610/20	600 LP	BV605
670/30	635 LP	660/20	630 LP	-
710/50	685 LP	710/50	685 LP	BV711
780/60	750 LP	780/60	750 LP	-
**488 nm**		**488 nm**		
488/10	No LP	488/10	No LP	SSC
530/30	505 LP	530/30	505 LP	FITC, BB515, VioBright FITC
710/50	685 LP	710/50	630 LP	PerCP-Cy5.5
**561 nm**		**561 nm**		
586/15	No LP	582/15	No LP	PE
610/20	600 LP	610/20	600 LP	PE-Dazzle
675/50	635 LP	685/35	635 LP	-
780/60	750 LP	780/60	750 LP	PE-Cy7, PC7
**640 nm**		**633 nm**		
670/30	No LP	670/14	No LP	APC
730/45	690 LP	730/45	690 LP	Fixable Viability Stain 700
780/60	750 LP	780/60	750 LP	APC-H7, APC-Alexa750

Supplementary Table 5. Target values for 7th peak of rainbow beads in fluorescent channels

Fluorochrome	Channel	Lower (-15%)	Target MFI	Upper (+15%)	recommendation
**BV421**	**V450**	100,452	**118,178**	135,905	EuroFlow
**BV510**	**V525**	93,871	**110,436**	127,002	EuroFlow
**BV605**	**V610**	47,731	**56,154**	64,577	In-house
**BV711**	**V710**	15,079	**17,740**	20,401	In-house
**FITC, BB515**	**B530**	28,752	**33,826**	38,900	EuroFlow
**PerCP-Cy5.5**	**B710**	66,846	**78,642**	90,438	EuroFlow
**PE**	**YG586**	32,381	**38,095**	43,809	EuroFlow
**PE-Dazzle**	**YG610**	178,500	**210,000**	241,500	In-house
**PE-Cy7**	**YG780**	8,316	**9,783**	11,250	EuroFlow
**APC**	**R670**	158,639	**186,634**	214,629	EuroFlow
**AF700**	**R730**	121,550	**143,000**	164,450	In-house
**APC-H7**	**R780**	64,194	**75,522**	86,850	EuroFlow

Spherotech Rainbow Calibration particles (8 peaks) 3.41µm; cat nr. RCP-30-5A, Lot No. EAG01

Supplementary Table 6: Clinical and immunological details of all patients

Patient	Age at inclusion (yr)	Age at diagnosis (yr)	Sex	Clinical Diagnosis	IgRT at time of sampling	anti-inflammatories within 4-6 months prior to sampling	B-cells/µl blood	T-cells/µl blood	IgG at inclusion (g/L)	IgA (g/L)	IgM (g/L)	Impaired vaccination responses	Infectious complications	Non-infectious complications	NIC at diagnosis
1	18	2/12	M	XLA	Y	N	**<1**	3148	N/A	N/A	N/A	N/A	sinusitis	none	N/A
2	21	3/12	M	XLA	Y	N	**<1**	1652	12*	**0.1**	**0.1**	N/A	pneumonia, bronchiectasis	none	N/A
3	22	3/12	M	XLA	Y	N	**<1**	1770	N/A	**<0.1**	**<0.1**	N/A	otitis, sinusitis, pneumonia	none	N/A
4	24	13	M	XLA	Y	N	**10**	1401	N/A	**0.1**	**0.1**	N/A	sinusitis, bronchiectasis, prostatitis	none	N/A
5	24	2	M	XLA	Y	N	**<0.1**	1497	N/A	**<0.1**	**<0.1**	N/A	otitis, sinusitis, pneumonia	leukemia^	N/A
6	26	1	M	XLA	Y	N	**<0.1**	2478	N/A	**<0.1**	**<0.1**	N/A	otitis, sinusitis, pneumonia, bronchiectasis	none	N/A
7	34	12	M	XLA	Y	N	**1**	**625**	N/A	**<0.1**	**<0.1**	N/A	otitis, sinusitis, pneumonia	none	N/A
8	49	12	M	XLA	Y	N	**3**	**801**	N/A	**0.1**	**0.1**	N/A	otitis, sinusitis, pneumonia, bronchiectasis	none	N/A
9	59	5	M	XLA	Y	HCT	**<0.1**	3141	N/A	**<0.1**	**<0.1**	N/A	otitis, sinusitis, pneumonia, asthma/COPD, bronchiectasis	none	N/A
10	24	18	F	agamma	N[#]	N	**10**	998	**0.6**	**0.1**	**0.1**	N/A	otitis, sinusitis, pneumonia	none	N/A
11	23	23	M	CVID	Y	N	446	1422	**4.9**	**0.3**	**0.3**	N/A	sinusitis	ITP/ AIHA/ Neutropenia/ Splenomegaly	Y
12	23	22	F	CVID	Y	N	215	1339	**4.4**	**0.2**	0.4	N/A	otitis, sinusitis	enteropathy	Y
13	24	24	M	CVID	N[#]	N	228	954	**1.1**	**<0.1**	**<0.1**	not done	otitis, sinusitis, pneumonia, VZV	none	N/A
14	24	23	F	CVID	Y	N	**17**	**736**	**<1.4**	**<0.2**	**<0.2**	not done	pneumonia, sinusitis	arthritis, enteropathy - duodenal and ilea	Y
15	25	12	M	CVID	Y	N	106	2562	**1.9**	**0.1**	**0.4**	N/A	otitis, sinusitis, pneumonia, bronchiectasis	tetralogy of Fallot/ VSD	Y
16	25	22	M	CVID	Y	N	230	1381	**2.2**	**<0.1**	**0.1**	N/A	sinusitis, pneumonia	arthritis	Y
17	26	15	M	CVID	Y	N	467	3355	**5.6**	**0.6**	**0.3**	pneumo	sinusitis	none	N/A
18	27	27	F	CVID	Y	N	166	2008	**3.9**	**0.7**	**0.3**	N/A	pneumonia, bronchitis	vitiligo	Y
19	28	28	M	CVID	N[#]	N	**56**	**819**	**3.3**	1	0.4	not done	bronchitis, pneumonia	pulmonary nodules and colitis	N
20	30	28	F	CVID	Y	N	1089	2953	**2.2**	**0.4**	**0.6**	N/A	sinusitis	cytopenia	Y
21	34	34	F	CVID	N[#]	N	**46**	1083	**<1**	**<0.1**	**<0.1**	N/A	otitis, sinusitis, pneumonia, bronchiectasis	GLILD	Y
22	35	30	F	CVID	Y	N	222	**646**	**2**	**0.1**	**0.1**	pneumo	sinusitis, giardia, asthma/COPD	enteropathy	N
23	37	24	M	CVID	Y	N	**78**	981	**4.1**	**0.1**	**0.4**	pneumo, Hib	otitis, sinusitis, pneumonia, systemic viral infection, giardia, asthma/COPD, bronchiectasis	granuloma, enteropathy	N
24	41	31	M	CVID	Y	–	**35**	1107	**2.6**	**0.2**	**0.2**	pneumo	sinusitis, pneumonia, systemic viral infection	granuloma, enteropathy	Y
25	43	35	F	CVID	Y	–	101	1242	**2**	**0.3**	**0.2**	pneumo	sinusitis, pneumonia	cytopenia	Y
26	44	36	F	CVID	Y	–	177	**614**	**2.7**	**0.1**	**0.1**	pneumo	sinusitis, pneumonia, giardia	splenomegaly, enteropathy	N

Patient	Age at inclusion (yr)	Age at diagnosis (yr)	Sex	Clinical Diagnosis	IgRT at time of sampling	anti-inflammatories within 4-6 months prior to sampling	B-cells/µl blood	T-cells/µl blood	IgG at inclusion (g/L)	IgA (g/L)	IgM (g/L)	Impaired vaccination responses	Infectious complications	Non-infectious complications	NIC at diagnosis
27	44	43	M	CVID	Y	–	**91**	834	**0.6**	**0**	**0.2**	N/A	sinusitis, pneumonia, bronchiectasis	splenomegaly, lymphadenopathy, granuloma, enteropathy, arthritis	Y
28	46	40	F	CVID	Y	–	180	**818**	**3**	**0.5**	0.4	pneumo	otitis, sinusitis, shingles	urticaria	N
29	46	41	M	CVID	Y	–	228	870	**0.6**	**0.1**	**0.1**	pneumo	otitis, sinusitis	none	N/A
30	47	40	F	CVID	Y	–	**84**	1210	**3.1**	**<0.1**	**<0.1**	N/A	sinusitis, bronchitis, conjunctivitis	autoimmune hypothyroidism, collagenous colitis	N
31	52	52	F	CVID	N[#]	PNL	138	1214	**4.8**	**0.8**	1.4	pneumo	otitis, sinusitis, pneumonia	none	N/A
32	53	53	F	CVID	Y	PNL	**96**	**678**	**3.8**	0.9	0.8	N/A	bronchitis, sinusitis	pericarditis	Y
33	54	16	F	CVID	N[#]	–	373	1473	**5.4**	1.6	**0.3**	pneumo	bronchiectasis, sinusitis	hypothyroidism	Y
34	54	54	F	CVID	Y	–	**95**	**631**	11*	**0.2**	0.4	N/A	viral pneumonia, otitis	none	N/A
35	54	45	M	CVID	Y	–	245	**775**	**2**	**<0.1**	**0.1**	N/A	sinusitis, pneumonia, asthma/COPD, bronchiectasis	none	N/A
36	54	46	M	CVID	Y	–	152	1040	**4.4**	**0.2**	**1**	pneumo, diphtheria, tetanus, Hib	sinusitis, pneumonia	solid organ malignancy	N
37	56	44	M	CVID	Y	PNL	**86**	**235**	11.7*	**<0.1**	**0.2**	N/A	pneumonia, hepatitis, colitis	AIHA, ITP, marked splenomegaly	Y
38	62	59	F	CVID	N	–	204	1229	**5.6**	1.3	**0.3**	pneumo	sinusitis	none	N/A
39	66	67	F	CVID	Y	PNL	161	**539**	**3.5**	**0.6**	**0.2**	N/A	bronchitis, bronchiectasis, asthma	none	N/A
40	73	66	F	CVID	Y	–	154	1345	**3.3**	**0.3**	**0.3**	N/A	sinusitis, asthma/COPD, bronchiectasis	none	N/A
41	77	68	F	CVID	Y	PNL	**70**	**553**	**4.2**	**0.8**	1.9	diphtheria	systemic viral infection, bronchiectasis, ILD	solid organ malignancy, auto-immunity	Y
42	82	68	M	CVID	Y	–	**13**	**727**	9.8*	**0.6**	**0.2**	N/A	otitis, sinusitis, pneumonia, bronchiectasis	cytopenia	N
43	27	4	M	HGG	Y	–	148	**742**	10.8*	1.8	1.3	N/A	pneumonia	none	N/A
44	29	21	F	HGG	Y	–	144	1982	**5.2**	**0.3**	0.5	normal	asthma/COPD	none	N/A
45	31	27	F	HGG	Y	–	361	983	**5.6**	**0.3**	1.3	normal	sinusitis, systemic viral infection, giardia	enteropathy	Y
46	40	40	F	HGG	N	–	202	1038	**5.2**	1.5	0.9	normal	sinusitis, pneumonia	none	N/A
47	43	42	F	HGG	Y	–	116	1554	**4.2**	3.2	0.6	pneumo	sinusitis, pneumonia	inflammatory tracheal stenosis	Y
48	45	45	F	HGG	Y	–	570	2029	11*	3.7	1.6	normal	otitis, sinusitis, pneumonia, asthma/COPD, pulmonary nocardia & aspergillus	none	N/A
49	52	50	F	HGG	N	–	193	1329	**5.2**	0.8	1.3	pneumo	sinusitis, pneumonia	none	N/A
50	52	47	M	HGG	Y	–	195	**473**	**3.2**	**0.3**	**0.2**	Hib	sinusitis, pneumonia	none	N/A
51	55	54	F	HGG	N[#]	–	215	1362	**4.1**	**0.4**	0.8	normal	pneumonia	inflammatory tenosynovitis	Y

Patient	Age at inclusion (yr)	Age at diagnosis (yr)	Sex	Clinical Diagnosis	IgRT at time of sampling	anti-inflammatories within 4-6 months prior to sampling	B-cells/µl blood	T-cells/µl blood	IgG at inclusion (g/L)	IgA (g/L)	IgM (g/L)	Impaired vaccination responses	Infectious complications	Non-infectious complications	NIC at diagnosis
52	60	59	F	HGG	Y	–	119	1408	**5.9**	**0.7**	1.3	not done	sinusitis, otitis, pneumonia	Hashimoto's thyroiditis	Y
53	67	61	F	HGG	Y	–	191	1844	**4.6**	**0.6**	2.9	diphtheria	sinusitis, pneumonia, asthma/COPD	enteropathy, autoimmunity	Y
54	68	68	F	HGG	N[#]	–	266	1157	**5.6**	1.7	0.9	pneumo	pneumonia, sinusitis	none	N/A
55	79	74	F	HGG	Y	–	**81**	**706**	**4**	1.1	1.5	pneumo	sinusitis, bronchiectasis, osteomyelitis, pertussis	breast carcinoma, polymyalgia, asthma	Y
56	42	35	M	IGSCD	N	–	477	1796	6.9	**0.6**	1.1	normal	bronchitis	none	N/A
57	19	19	F	SpAD	N[#]	–	**92**	2090	11.1	**<0.1**	0.9	pneumo	sinusitis, bronchitis, tonsillitis	none	N/A
58	21	20	M	SpAD	N	–	192	1209	9.4	0.9	1.1	pneumo	sinusitis	none	N/A
59	41	35	M	SpAD	Y	CT	236	1894	8.2*	2.1	**0.3**	pneumo	otitis, sinusitis, asthma/COPD	none	N/A
60	57	58	F	SpAD	N[#]	HCQ	252	1087	<u>32*</u>	3	0.5	pneumo	pneumonia	SLE/Sjögren's	Y
61	66	65	F	SpAD	N[#]	–	323	957	10.3	**<0.1**	0.9	poor	pneumonia, bronchitis	none	N/A
62	72	72	F	SpAD	N[#]	–	119	1413	12	1.8	0.7	poor	pneumonia, bronchiectasis, MAC, pseudomonas	none	N/A

Values below normal range are depicted in **bold** font and above normal range <u>underlined</u>. Reference ranges: IgG, 6.1-16.2g/L; IgA, 0.85-4.99g/L; IgM, 0.35-2.42g/L; B cells, 97-614 cells/µl; T cells, 823-2430 cells/µl. (Reference ranges represent the 5th and 95th percentiles from our healthy controls).

[#] Ig replacement commenced after inclusion in this study; * IgG measurement after start of Ig replacement therapy; ^leukemia diagnosed post inclusion (van Zelm et al., 2019); agamma, agammaglobulinemia; CVID, common variable immunodeficiency; HGG, hypogammaglobulinemia; IGSC, IgG subclass deficiency; SpAD, specific antibody deficiency; XLA, X-linked agammaglobulinemia (genetically diagnosed see ***Supplementary Table 6),*** IgRT, immunoglobulin replacement therapy; CT, cortisone; HCT, hydrocortisone; HCQ, hydroxychloroquine; PNL, prednisolone; MAC, Mycobacterium Avium complex; pneumo, pneumococcal; SLE, systemic lupus erythematosus.

Supplementary Table 7: Molecular diagnosis of XLA patients.

Family	Patient	Gene	Mutation (DNA)	location	Effect (protein)
A	1	*BTK*	c.1257delG	Exon 14	p.G419GfsX11
	2	*BTK*	c.1257delG	Exon 14	p.G419GfsX11
	3	*BTK*	c.1257delG	Exon 14	p.G419GfsX11
	4	*BTK*	c.1257delG	Exon 14	p.G419GfsX11
	6	*BTK*	c.1257delG	Exon 14	p.G419GfsX11
B	5 *	*BTK*	c.1908+1G>C	Exon 18	p.V626_E636del
C	7	*BTK*	c.862C>T	Exon 10	p.R288W
D	8	*BTK*	c.1559G>A	Exon 15	p.R520Q
E	9	*BTK*	c.1787+71C>T	Intron 16	p.A523V fsX5

*Patient presented with B-ALL 6 months after inclusion in this study and was reported in (van Zelm et al., 2019)

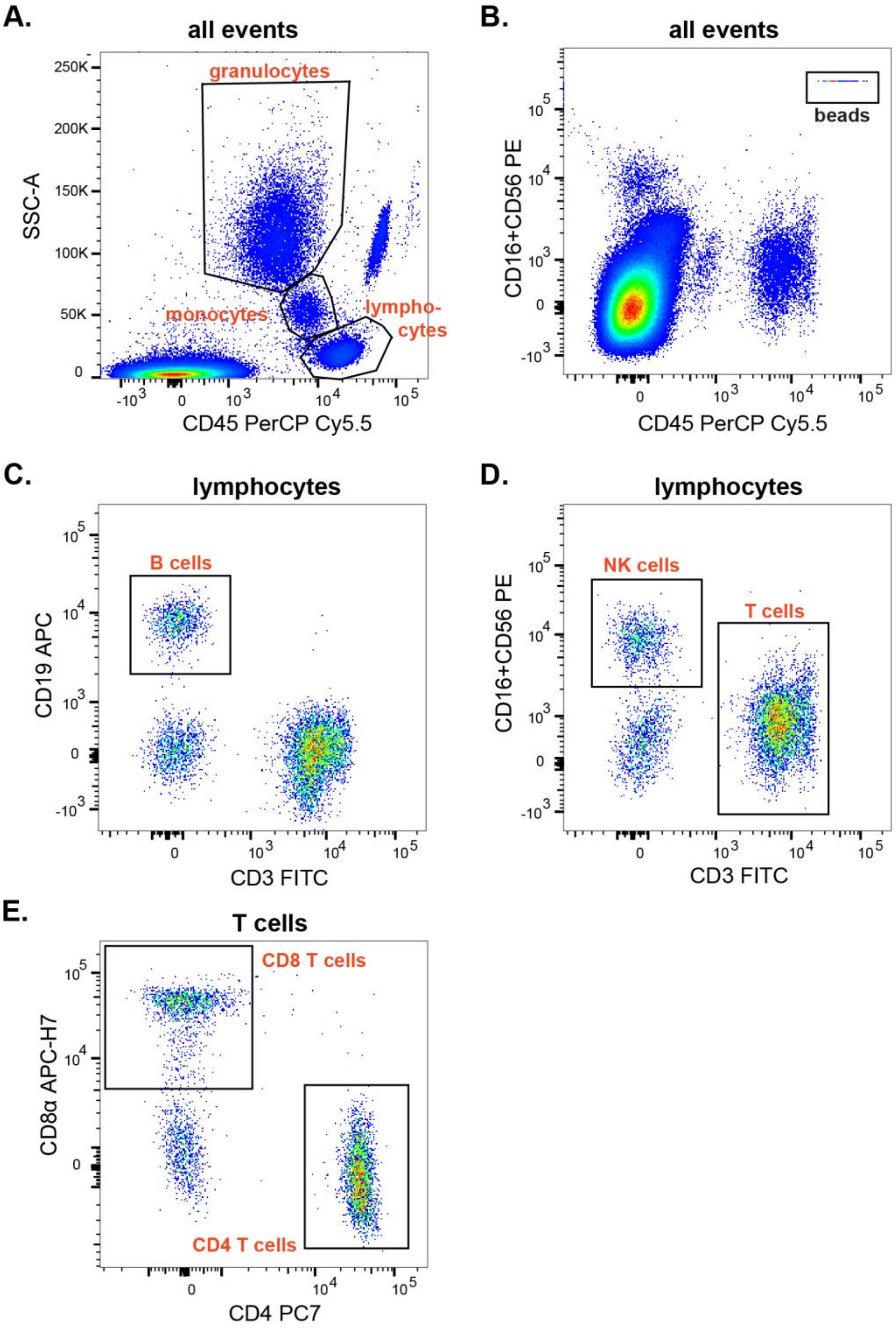

Supplementary Figure 1. Gating strategy for TruCount tube. A. Gating of major leukocyte subsets, and **B.** beads within all events. **C.** Gating of $CD19^+$ B cells within the lymphocyte gate. **D.** Gating of $CD16/56^+$ NK cells and $CD3^+$ T cells within the lymphocyte gate. **E.** Gating of CD4 T cells and CD8 T cells within the total T cell gate.

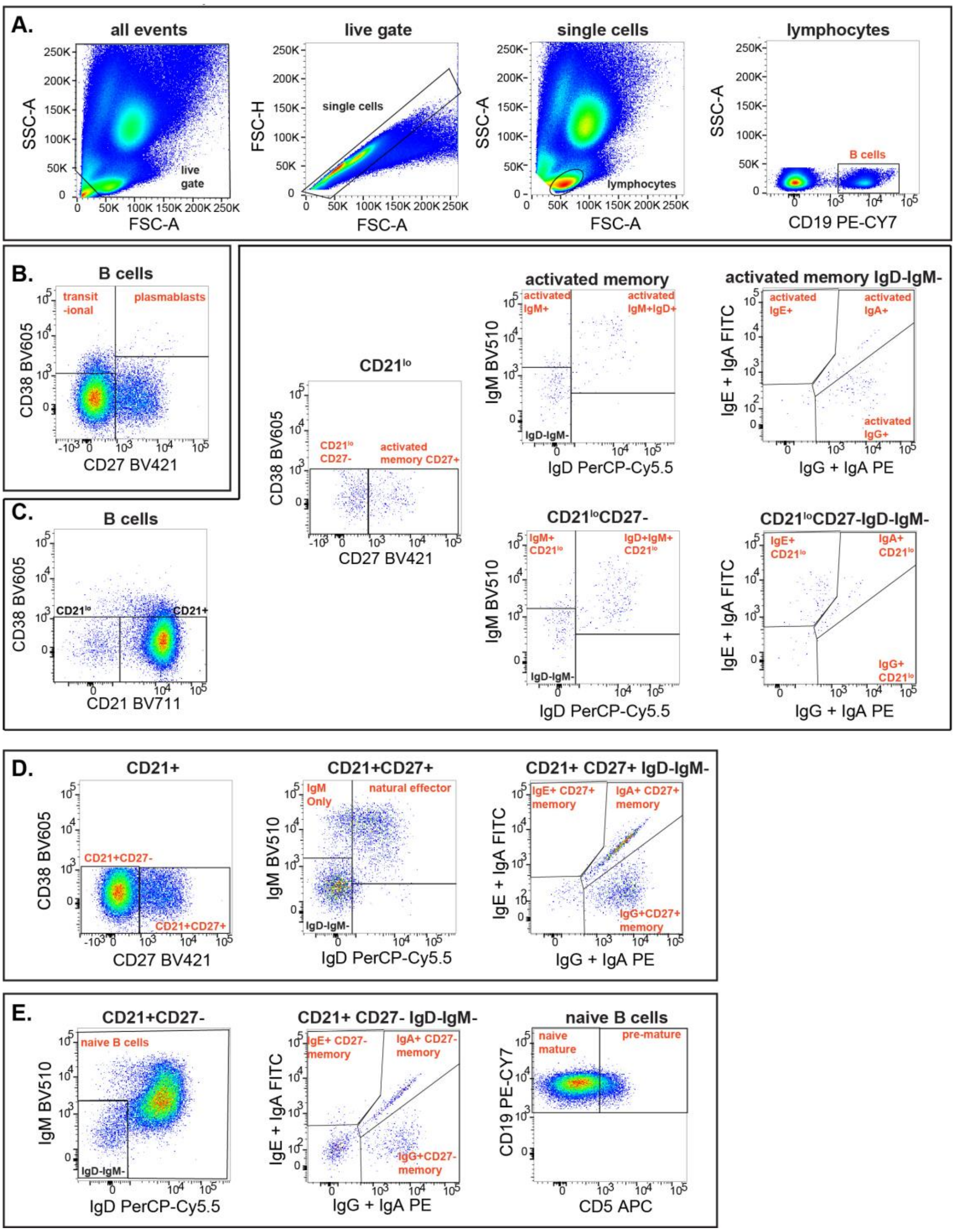

Supplementary Figure 2. Gating strategy for the B-cell tube. A. Gating strategy to define total $CD19^{+}$ B cells. (**B**) Transitional B cells and plasmablasts are delineated from B cells. **C.** In parallel, B cells are gated based on dim expression CD38 and divided in to $CD21^{lo}$ or $CD21^{+}$ B cells. $CD21^{+}$ gated in **C.** are subdivided into **D.** $CD21^{+}CD27^{+}$ and **E.** $CD21^{+}CD27^{-}$. **D.** $CD21^{+}CD27^{+}$ cells are assessed according to their expression of IgD and IgM to delineate IgM only and natural effector cells. The $IgD^{-}IgM^{-}$ cells are further characterized according to IgA, IgE and IgG expression in to $IgE^{+}CD27^{+}$ memory, $IgA^{+}CD27^{+}$ memory and $IgG^{+}CD27^{+}$ memory. **E.** $CD21^{+}CD27^{-}$ cells are subdivided according to CD27 expression in to naive B cells and $IgD^{-}IgM^{-}$. The $IgD^{-}IgM^{-}$ are subsequently assessed for IgA, IgG and IgE expression to delineate $IgE^{+}CD27^{-}$ memory, $IgA^{+}CD27^{-}$ memory and $IgG^{+}CD27^{-}$ memory subsets. Naive B cells are assessed for CD5 expression to delineate pre-naive and naive mature B cells.

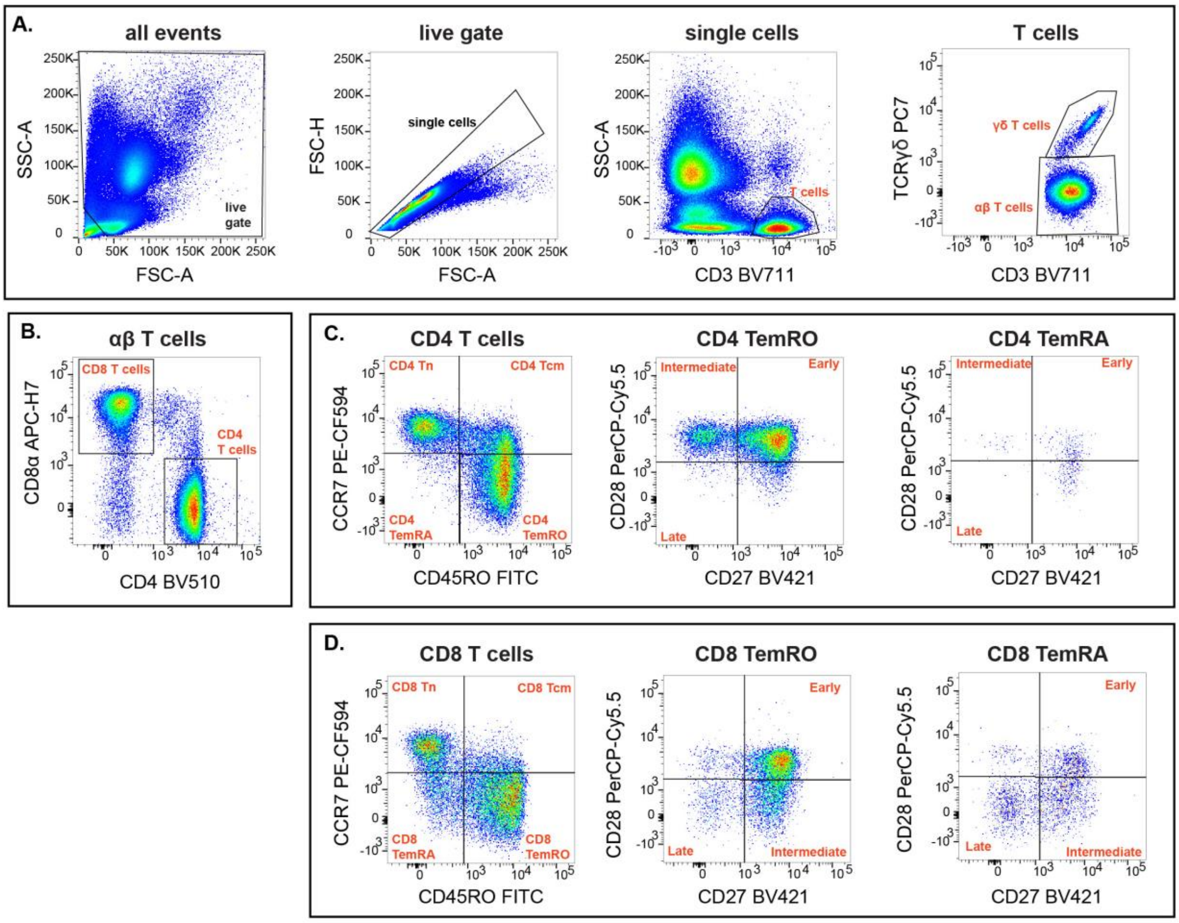

Supplementary Figure 3. Gating strategy for T Effector tube. A. Gating strategy to define total, γδ T cells and αβ T cells, **B.** αβ CD4 and CD8 T cells. **C.** Gating of CD4 T cells into naive (CD4 Tn) and memory (CD4 Tcm, TemRO and TemRA) T cell subsets, followed by subsetting CD4 TemRO and TemRA into early ($CD27^{+}CD28^{+}$), intermediate ($CD27^{-}CD28^{+}$) and late ($CD27^{-}CD28^{-}$) stage. **D.** Gating of CD8 T cells into naive (CD8 Tn) and memory (CD8 Tcm, TemRO and TemRA) T cell subsets, followed by subsetting CD8 TemRO and TemRA into early ($CD27^{+}CD28^{+}$), intermediate ($CD27^{+}CD28^{-}$) and late ($CD27^{-}CD28^{-}$) stage.

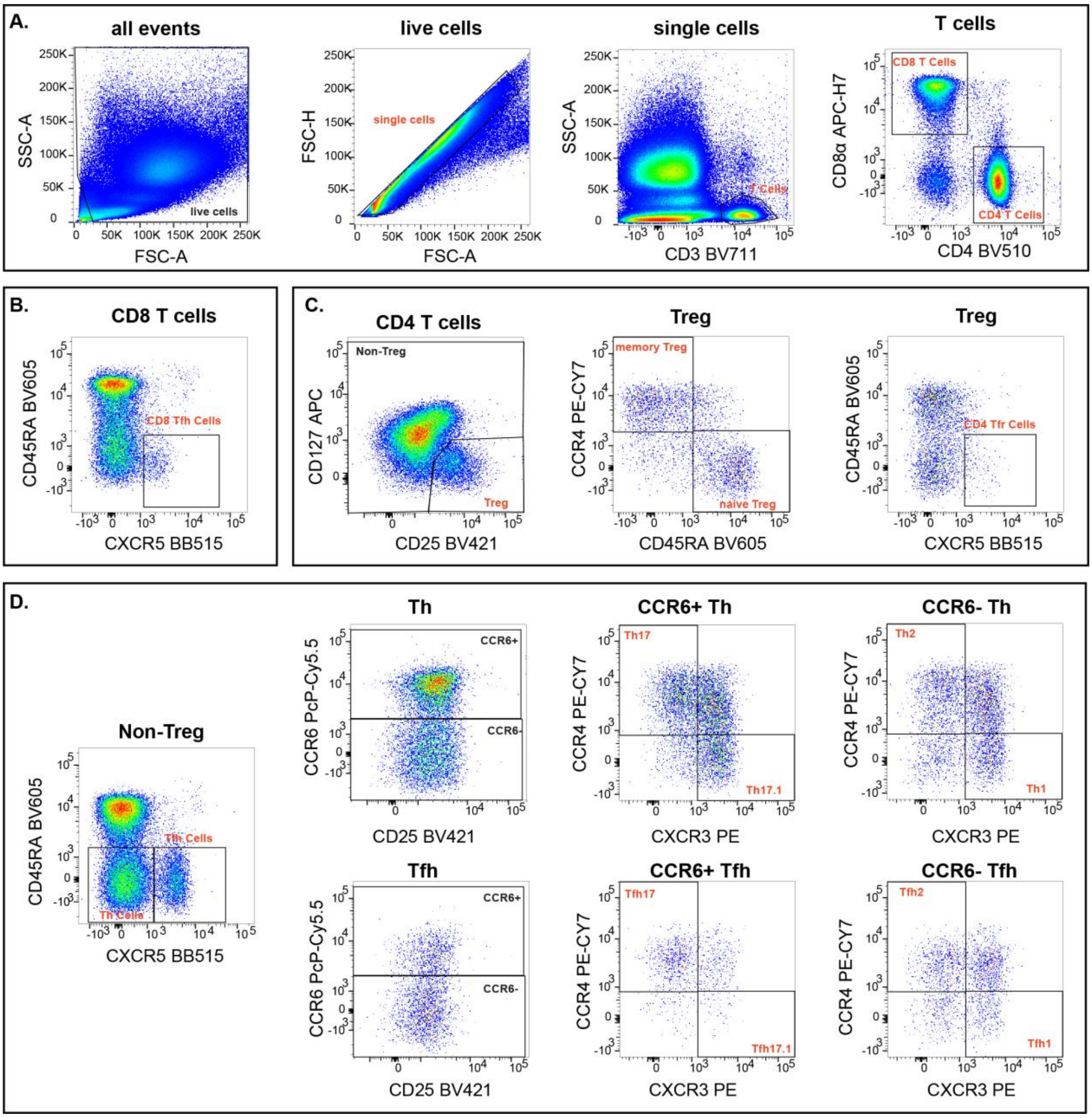

Supplementary Figure 4. Gating strategy for T helper cell tube. A. Gating strategy to define total CD4 and CD8 T cells. **B.** CD8 Tfh T cells are gated within the CD8 T cell gate defined in A. **C.** Tregs ($CD25^{hi}CD127^{lo}$) and non-Tregs are defined within the CD4 T cell gate. Tregs are further separated into naive and memory Tregs and Tfr. **D.** Non-Tregs are subdivided into Th ($CXCR5^{-}CD45RA^{-}$) and Tfh ($CXCR5^{+}CD45RA^{-}$). Th cells are further split into $CCR6^{+}$ and $CCR6^{-}$ with each population further delineated using CXCR3 and CCR4 expression, resulting in the gating of four Th subpopulations Th1, Th2, Th17 and Th17.1. Tfh are subdivided in to CCR6 + and CCR6- with each of these populations further subdivided to yield Tfh1, Tfh2, Tfh17 and Tfh17.1.

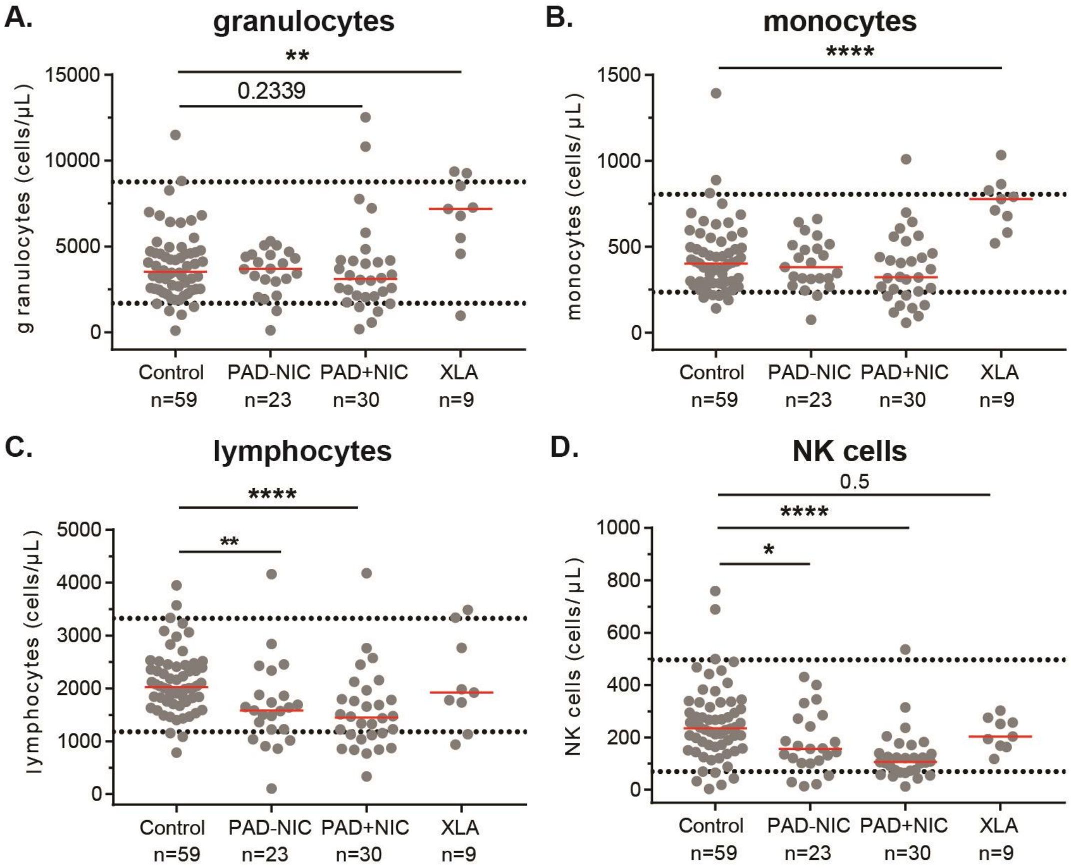

Supplementary Figure 5. Lymphocyte and NK cell numbers are significantly reduced in PAD patients. Absolute numbers of **A.** Granulocytes, **B.** Monocytes, **C.** Lymphocytes and **D.** NK cells. Gating strategy is outlined in ***Suppl Figure 1*** and ***Suppl Table 3***. Horizontal dotted lines represent the 5th and 95th percentiles from the healthy control group. PAD, predominantly antibody deficiency; NIC, non-infectious complications. Statistics were performed using the Kruskall-Wallis test, followed by Mann-Whitney tests for pairwise comparisons. *P<0.5, **P<0.01, ***P<0.001, and ****P<0.0001.

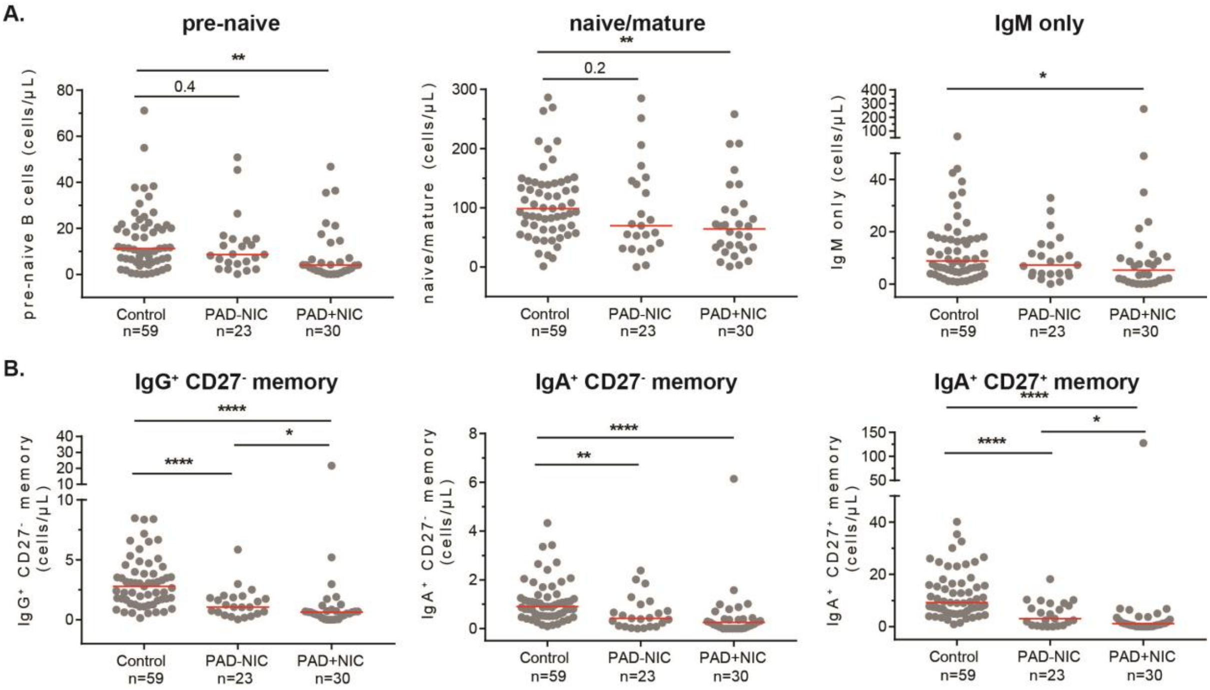

Supplementary Figure 6. Reduced naive and memory B cells in PAD patients. Absolute numbers of **A.** pre-naive, naive mature and IgM memory, **B**. IgG$^+$ CD27$^-$, IgA$^+$ CD27$^-$ and IgA$^+$ CD27$^+$ memory B cells. Subsets delineated as in ***Suppl Figure 2*** and ***Suppl Table 3***. PAD, predominantly antibody deficiency; NIC, non-infectious complications. Statistics were performed using a two-way ANOVA, followed by a Mann-Whitney test. *P<0.5, **P<0.01, ***P<0.001, and ****P<0.0001.

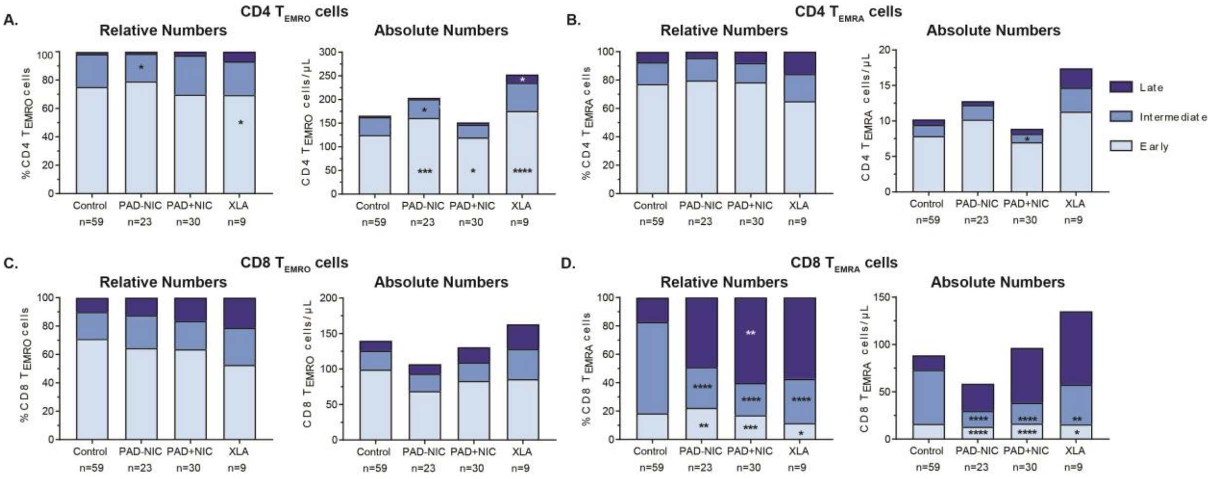

Supplementary Figure 7. Altered early, intermediate and late T_{EMRO} and T_{EMRA} cells in PAD patients. Relative and absolute numbers of CD4 (**A**) TemRO and (**B**) TemRA subsets: early ($CD27^{+}CD28^{+}$), intermediate ($CD27^{-}CD28^{+}$) and late ($CD27^{-}CD28^{-}$) stages. Relative and absolute numbers of CD8 (C) TemRO and (D) TemRA subsets: early ($CD27^{+}CD28^{+}$), intermediate ($CD27^{+}CD28^{-}$) and late ($CD27^{-}CD28^{-}$) stages. Subsets delineated as in ***Suppl Figure 3*** and ***Suppl Table 3***. PAD, predominantly antibody deficiency; NIC, non-infectious complications. Statistics were performed using a two-way ANOVA, followed by a Mann-Whitney test. *P<0.5, **P<0.01, ***P<0.001, and ****P<0.0001.
